# Attenuated transcriptional response to pro-inflammatory cytokines in schizophrenia hiPSC-derived neural progenitor cells

**DOI:** 10.1016/j.bbi.2022.06.010

**Published:** 2022-10

**Authors:** Anjali Bhat, Haritz Irizar, Amalie C.M. Couch, Pooja Raval, Rodrigo R.R. Duarte, Lucia Dutan Polit, Bjorn Hanger, Timothy Powell, P.J. Michael Deans, Carole Shum, Roland Nagy, Grainne McAlonan, Conrad O. Iyegbe, Jack Price, Elvira Bramon, Sagnik Bhattacharyya, Anthony C. Vernon, Deepak P. Srivastava

**Affiliations:** a Department of Basic & Clinical Neuroscience, Institute of Psychiatry, Psychology & Neuroscience, King’s College London, London, UK; b MRC Centre for Neurodevelopmental Disorders, King’s College London, UK; c Division of Psychiatry, University College London, London, UK; d Wellcome Centre for Human Neuroimaging, University College London, London, UK; e Department of Social, Genetic & Developmental Psychiatry, Institute of Psychiatry, Psychology & Neuroscience, King's College London, London, UK; f Department of Forensic and Neurodevelopmental Sciences, Institute of Psychiatry, Psychology & Neuroscience, King's College London, London, UK; g Department of Psychosis Studies, Institute of Psychiatry, Psychology & Neuroscience, King's College London, London, United Kingdom; h Icahn School of Medicine, Mount Sinai Hospital, NY, USA; i Department of Medicine, Weill Cornell Medical College, Cornell University, NY, USA

**Keywords:** Maternal immune activation, Inflammation, Cytokine, IL-1β, IFNγ, Differential gene expression, Neurotransmission, Prenatal development, Neurodevelopment

## Abstract

Maternal immune activation (MIA) during prenatal development is an environmental risk factor for psychiatric disorders including schizophrenia (SZ). Converging lines of evidence from human and animal model studies suggest that elevated cytokine levels in the maternal and fetal compartments are an important indication of the mechanisms driving this association. However, there is variability in susceptibility to the psychiatric risk conferred by MIA, likely influenced by genetic factors. How MIA interacts with a genetic profile susceptible to SZ is challenging to test in animal models. To address this gap, we examined whether differential gene expression responses occur in forebrain-lineage neural progenitor cells (NPCs) derived from human induced pluripotent stem cells (hiPSC) generated from three individuals with a diagnosis of schizophrenia and three healthy controls. Following acute (24 h) treatment with either interferon-gamma (IFNγ; 25 ng/μl) or interleukin (IL)-1β (10 ng/μl), we identified, by RNA sequencing, 3380 differentially expressed genes (DEGs) in the IFNγ-treated control lines (compared to untreated controls), and 1980 DEGs in IFNγ-treated SZ lines (compared to untreated SZ lines). Out of 4137 genes that responded significantly to IFNγ across all lines, 1223 were common to both SZ and control lines. The 2914 genes that appeared to respond differentially to IFNγ treatment in SZ lines were subjected to a further test of significance (multiple testing correction applied to the interaction effect between IFNγ treatment and SZ diagnosis), yielding 359 genes that passed the significance threshold. There were no differentially expressed genes in the IL-1β-treatment conditions after Benjamini-Hochberg correction. Gene set enrichment analysis however showed that IL-1β impacts immune function and neuronal differentiation. Overall, our data suggest that a) SZ NPCs show an attenuated transcriptional response to IFNγ treatment compared to controls; b) Due to low IL-1β receptor expression in NPCs, NPC cultures appear to be less responsive to IL-1β than IFNγ; and c) the genes differentially regulated in SZ lines – in the face of a cytokine challenge – are primarily associated with mitochondrial, “loss-of-function”, pre- and post-synaptic gene sets. Our findings particularly highlight the role of early synaptic development in the association between maternal immune activation and schizophrenia risk.

## Highlights

• Human cortical NPCs show a significant transcriptional response to IFNγ treatment.
• This response is attenuated in cortical NPCs from schizophrenia patients.
• The abnormal transcriptional response is driven by mitochondrial and synaptic genes.
• Cortical NPCs do not show significant transcriptional response to IL-1β treatment.

## 1 Introduction

Activation of the maternal immune response during pregnancy is a known risk factor for neurodevelopmental disorders – especially autism and schizophrenia – in the offspring (Estes and McAllister, 2016, Warre-Cornish et al., 2020, Byrne et al., 2007, Kepinska et al., 2020, Meyer, 2019). Although the precise molecular mechanisms driving this association remain unclear, exposure of the developing fetal brain to pro-inflammatory cytokines is a promising candidate for study (Warre-Cornish et al., 2020, Garay et al., 2013, Gilmore et al., 2004, Gilmore et al., 2005). Cytokines are cell signalling proteins that help immune cells to form coordinated responses to infection. Whilst their function in the peripheral immune system is well documented, there is growing evidence that cytokines also play an important role in brain development and that maternally-derived cytokines can affect the developing foetal brain (Warre-Cornish et al., 2020, Garay et al., 2013, Gilmore et al., 2004, Gilmore et al., 2005). Emerging evidence from human studies suggest that elevated levels of canonical pro-inflammatory cytokines such as interferon gamma (IFNγ) (Lesh et al., 2018, Warre-Cornish et al., 2020) and interleukin-1 beta (IL-1β) (Gilmore et al., 2004, Crampton et al., 2012) can be detected in the plasma of individuals with a diagnosis of schizophrenia (Goldsmith et al., 2016, Lesh et al., 2018). Furthermore, serum levels of IL-1β are elevated in the mothers of offspring who later develop psychosis (Allswede et al., 2020). Animal models of maternal immune activation (MIA) also provide evidence for elevated IFNγ and IL-1β levels in maternal serum, as well as the serum and brains of fetuses (Garay et al., 2013, Arrode-Bruses and Bruses, 2012). Moreover, in mice exposed to MIA, offspring who are susceptible (those that show, as adults, deficits in social and cognitive behaviours relevant for schizophrenia) have elevated plasma levels of IL-1β (among other cytokines), as compared to control mice and MIA-exposed mice that are resilient to MIA i.e. they do not show these abnormal behaviours (Mueller et al., 2021).

However, the impact and outcome following prenatal immune activation is heterogeneous between individuals (Meyer, 2019, Mueller et al., 2021, Carlezon et al., 2019). As mentioned above, the work of Mueller and colleagues (2021) highlights the existence of subgroups of MIA-exposed offspring that show dissociable behavioural, transcriptional, neuroimaging, and immunological profiles (Mueller et al., 2021, Estes et al., 2020). This is consistent with epidemiological studies in human cohorts: not all foetuses exposed to MIA will go on to develop schizophrenia (Estes and McAllister, 2016, Brown, 2006, Brown et al., 2004). It is therefore likely that MIA interacts with other factors, such as genetic background, to modulate the risk of developing schizophrenia or other outcomes. Indeed, the two-hit model of schizophrenia suggests that an amalgam of genetic risk and environmental insult is necessary to alter neurodevelopment enough to ultimately precipitate the symptoms of the disorder (Feigenson et al., 2014, van Os et al., 2008, Bayer et al., 1999).

Importantly, if there are individual differences in human responses to immune activation due to genetic variability, there will certainly be differences in such responses between human and animal systems, given that they are even more genetically divergent. Although animal studies have provided important mechanistic insights, interactions between schizophrenia genetic burden and MIA cannot be fully recapitulated by rodent models: species differences in gene expression cannot be discounted (Leenaars et al., 2019, Pound and Ritskes-Hoitinga, 2018, Masopust et al., 2017, Canetta and Kellendonk, 2018). It is therefore important to test the impact of gene-environment interactions in human model systems, such as human induced pluripotent stem cells (hiPSCs). These pluripotent cells are generated by the reprogramming of somatic cells, such as hair keratinocytes and skin fibroblast biopsies, collected from patient cohorts or healthy controls (Takahashi and Yamanaka, 2006, Aasen and Izpisua Belmonte, 2010, Petit et al., 2012). The resulting hiPSCs can then be differentiated into multiple relevant cell types that retain the genetic make-up of the donor (Brennand et al., 2015, Brennand and Gage, 2012) – including neural progenitor cells (NPCs), early precursors to neurons which are highly prevalent in the fetal brain (Martinez-Cerdeno and Noctor, 2018). There is evidence that hiPSC-derived NPCs closely resemble fetal brain tissue, recapitulating the neurodevelopmental hallmarks of the late first trimester/early second trimester stage (Warre-Cornish et al., 2020, Brennand et al., 2015, Brennand and Gage, 2012, Shum et al., 2020, Adhya et al., 2020, Kathuria et al., 2018). These facets make hiPSC-NPCs uniquely placed to model human fetal neurodevelopmental mechanisms and gene-environment interactions in vitro (Adhya et al., 2020, Hoffman et al., 2017).

Previous work from our group demonstrated that transient IFNγ treatment (24 hr) of hiPSC-NPCs from healthy controls increases neurite outgrowth (a cellular phenotype associated with neurodevelopmental disorders) and disproportionately alters the expression of genes associated with schizophrenia and autism (Warre-Cornish et al., 2020). The aim of the current study is therefore to understand how specific cytokines (IFNγ and IL-1β) implicated in the association between MIA and schizophrenia risk, influence transcriptional responses in NPCs derived from individuals with, or without a diagnosis of schizophrenia. We used cortical NPCs with forebrain identity, as there is extensive evidence of prefrontal cortical abnormalities in patients with schizophrenia, and experiments in animal models have shown that exposure to inflammatory cytokines alters proliferation and differentiation of neural progenitors (Arrode-Bruses and Bruses, 2012, Baines et al., 2020). The concentrations of IFNγ and IL-1β used in this study are based on those used in (Warre-Cornish et al., 2020), as they were effective in eliciting a response in NPCs that can be measured at a single time-point. These concentrations were more acute than those that would be observed *in vivo* in an MIA model system – but note that in this study we are not developing a model system of MIA but investigating whether the transcriptional response to cytokines that have previously been seen to play a role in MIA differs when this occurs on the genetic background associated with schizophrenia. This is an exploratory study of the transcriptional responses to IFNγ and IL-1β, with the aim of narrowing down on specific genes and key pathways that they influence in fetal-stage NPCs (of the sort that could be exposed to MIA). We hypothesise that NPCs derived from patients with schizophrenia will respond differently to IFNγ and IL-1β compared to cells from healthy donors. If so, this may shed light on the mechanisms by which maternal immune activation increases the risk of developing schizophrenia.

## 2 Materials and methods

### 2.1 Participants

This study included hiPSC lines derived from six participants: three individuals with a diagnosis of SZ (cell lines SCZ_138, SCZ_044 and SCZ_115) (Supplementary Fig. 1) and three healthy donors with no history of psychiatric illness (cell lines M1_CTR, M2_CTR, M3_CTR – previously described in (Shum et al., 2020, Adhya et al., 2020). Participants were recruited as part of the Patient iPSCs for Neurodevelopmental Disorders (PiNDs) study (REC No 13/LO/1218). Participants with a diagnosis of schizophrenia were recruited at the Maudsley Hospital, London. The collection of data used for this research was approved by the NHS Research Ethics Committee at the South London and Maudsley (SLaM) NHS Research and Development Office. All participants gave written informed consent before contributing to the study. A diagnosis of schizophrenia was established based on International Classification of Diseases (10th revision) (World Health O, 2004) with the diagnosis (code F20), assessed using the Operational Criteria checklist (McGuffin et al., 1991) by a psychiatrist on the basis of information recorded by the clinical team following psychiatric interview. Healthy, unaffected individuals were selected as controls on the basis of having no history of psychiatric disorders (Adhya et al., 2020).

### 2.2 Reprogramming of keratinocytes

Hair root samples were collected by plucking occipital scalp hair (∼10 + roots per participant) and submerging these in Mouse Embryonic Fibroblast medium containing 50 µg/mL Gentamycin and 15 mM HEPES buffer (Gibco). The roots were then transferred to Geltrex™-coated 4-well plates (ThermoFisher), and outgrowth promoted, by supplementing with hair medium (Dulbecco’s Modified Eagle’s Medium (DMEM) Advanced (Sigma Aldrich), GlutaMAX™ (ThermoFisher), 10% FBS (Clonetech), HEPES buffer and Gentamycin), to establish primary keratinocytes. The keratinocytes were subsequently reprogrammed into human induced pluripotent stem cell (hiPSC) lines. This transformation was induced by introducing Sendai viruses encoding Yamanaka Factors (human OCT4, SOX2, KLF4 and C-MYC), using a CytoTune-iPS 2.0 Sendai expressing Reprogramming Kit (ThermoFisher, A16517). The treated keratinocytes were plated onto an irradiated MEF feeder layer (Millipore) and supplemented Epilife medium. After ten days, Epilife medium was exchanged for hES medium, which was comprised of KO-DMEM/F12 supplemented with 20% knock-out serum, non-essential amino acids, Glutamax, b-mercaptoethanol (all from Life Technologies) and bFGF (10 ng/mL; Peprotech). After two more weeks, reprogrammed colonies were selected and plated on Nunc multi-plates (Thermo Scientific) coated with Geltrex (Life technologies) and supplemented with E8 media (Life Technologies).

### 2.3 Maintenance of hiPSCs

The successfully reprogrammed hiPSCs were incubated in hypoxic conditions (5% CO2, 5% O_2_) at 37 °C and maintained in StemFlex™ media (Gibco) on 6-well NUNC™ plates (ThermoFisher) coated with Geltrex™ (ThermoFisher). Cells were passaged (at a ratio between 1:6 and 1:18) upon reaching 60–70% confluency. During passage, cells were washed with room temperature Hank’s Balanced Salt Solution (HBSS) and incubated at 37 °C with Versene (EDTA) solution (Lonza) for 3–5 min, then replated in new Geltrex™-coated NUNC™ plates.

### 2.4 Directed differentiation of hiPSCs

The six hiPSC lines used in this study were then differentiated into forebrain cortical neural progenitor cells (NPCs) by dual SMAD inhibition (Shum et al., 2020, Adhya et al., 2020). In preparation for neuralisation, hiPSCs were passaged onto 6-well NUNC™ plates coated with Geltrex™ at a 3:2 ratio and maintained under hypoxic conditions for ∼ 24–48 hrs until they approached 100% confluence. Directed differentiation was then initiated by changing StemFlex™ medium to neuralisation medium containing N2:B27 (N2 medium and B27 medium at a 1:1 ratio) supplemented with 100 nM LDN193189 (Sigma Aldrich) and 10 µM SB431542 (Sigma Aldrich) for dual SMAD inhibition. N2 medium consisted of DMEM/F12 (Dulbecco’s Modified Eagle’s Medium/Nutrient Mixture F12 Ham; Sigma Aldrich), supplemented with 1X GlutaMAX™ and 1X N2 supplement (ThermoFisher). B27 medium consisted of Neurobasal® medium (ThermoFisher), 1X GlutaMAX™ (ThermoFisher) and 1X B27 supplement without vitamin A (ThermoFisher).

The neuralised cells were then incubated under normoxic conditions (37 °C, 5% CO_2_, 20% O_2_). Neuralisation medium was replenished every 24 h from day 0 to day 7. At the end of this 7-day neuralisation period, neuralisation medium was replaced with N2:B27 (without inhibitors), which was replenished every 24 h from day 8 onwards. The neuralised cells were passaged four times: on day 7, day 12, day 15/16 and day 20/21. The passage procedure was, briefly, as follows: cells were washed with room temperature HBSS (ThermoFisher) and treated with Accutase (ThermoFisher) and incubated for 3–4 min at 37 °C. The cells were then collected with the Accutase and mixed with room temperature DMEM/F12 (at a 2:1 ratio) and centrifuged at 1250 RPM for two minutes to separate the cells and Accutase. Cells were plated on new 6-well NUNC™ plates coated with Geltrex™. Passaging ratios were 1:1 for neural passaging 1 and 2, and 2:3 for neural passaging 3. To enhance cell survival, 10 µM protein kinase (ROCK) inhibitor (Sigma Aldrich), was added for 24 h with the plating medium at each neural passage. After neural passage 3, cells were frozen in 10% DMSO (dimethyl sulfoxide). Cryovials were stored at −80 °C for 24–48 h in Mr. Frosty containers (to control freezing rate) before being transferred to liquid nitrogen.

For the final stages of neural passaging, cryovials were thawed in a 37 °C water bath for 1 min. The cell suspension was transferred to a 15 mL tube containing DMEM/F12 and centrifuged at 1250 RPM for 2 min. The cell pellet was resuspended in 3 mL of N2:B27 supplemented with 10 µM ROCK inhibitor and plated in Geltrex™-coated 6-well NUNC™ plates. From this point on, the following inhibitors were added to the NPC media (to make N2:B27-FGF): 10 ng/mL bFGF (basic Fibroblast Growth Factor; Peprotech), 100 μM β-mercaptoethanol (Life Technologies), 5 μg/mL insulin (Life Technologies), 1X non-essential amino acids (Life Technologies), 200 μM ascorbic acid (Sigma Aldrich). The cells were then expanded at a 1:3 ratio (in 2–5 neural passages) to prepare three wells of each line (one for each experimental condition).

Successful reprogramming of hiPSCs was validated as described in previous studies (Shum et al., 2020, Kathuria et al., 2018, Cocks et al., 2014). Pluripotency of all hiPSCs was confirmed by immunocytochemistry, differentiation of embryoid bodies into the three characteristic germ layers (Sheridan et al., 2012, Chambers and Tomlinson, 2009, International Stem Cell Initiative, 2007, Boulting et al., 2011) (Supplemental Fig. 1), and PluriTest analysis of Illumina HT12v4 transcriptome array data (https://www.pluritest.org) (Muller et al., 2011). Alkaline phosphatase activity was further used to assess the pluripotency of hiPSCs using an alkaline phosphatase expression kit (Milipore). Genome integrity of hiPSC lines was assessed by an Illumina Human CytoSNP-12v2.1 beadchip array and analysed using KaryoStudio software (Illumina, San Diego, CA).

### 2.5 Acute treatment with pro-inflammatory cytokines

NPCs were treated for ∼ 24 h in three treatment conditions: IFNγ, IL-1β or vehicle. Media was fully removed and replaced with 3 mL per well of treatment media (N2:B27-FGF, supplemented as follows). IFNγ wells were treated with 25 ng/μL IFNγ (Abcam); the IL-1β wells with 10 ng/μL IL-1β (Abcam), as in (Warre-Cornish et al., 2020); and the control wells with vehicle (unsupplemented N2:B27-FGF media). After 24 h, cells were lysed and collected in TRIzol® reagent (Thermo Fisher) and rapidly frozen on dry ice. The frozen samples were stored at −80 °C until RNA extraction.

### 2.6 RNA extraction and sequencing

RNA was extracted from the eighteen samples in two batches (to ensure durations of exposure of each sample to extraction reagents were well controlled). Both batches of extractions were conducted on the same day, by the same experimenter. The batches were randomised for experimental group (batch 1: lines M1_CTR, M2_CTR, SCZ_138; batch 2: lines M3_CTR, SCZ_044, SCZ_115), using the RNeasy Plus Mini Kit (QIAGEN), according to the manufacturer’s instructions. Extracted RNA was sent for sequencing at GENEWIZ® Ltd. Strand-specific, paired-end RNA sequencing with Poly(A) selection was performed using the Illumina® NovaSeq platform, at a read length of ∼ 30 million reads per sample.

### 2.7 Quality control of RNA sequence and gene expression data

Initial quality control checks of raw RNA sequence data were conducted using the FastQC software from Babraham Bioinformatics (www.bioinformatics.babraham.ac.uk/projects/fastqc). Sequence reads were then aligned to the latest version of the human reference genome (hg38) using the STAR (Spliced Transcripts Alignment to a Reference) alignment tool (Baruzzo et al., 2017). The number of reads mapped onto each gene in Ensembl’s gene annotations for hg38 (version 99) was counted using FeatureCounts (Liao et al., 2014). We plotted the distribution of log_10_-transformed counts-per-million (CPMs) and, by visual inspection, set a threshold of log_10_CPM = 0.6 for filtering out lowly expressed genes in order to minimise technical noise and reduce the multiple-testing burden (Supplementary Fig. 2). After applying that threshold, 15,060 out of 60,642 genes were left for downstream analysis. TMM (trimmed mean of M−values)-normalization (Robinson and Oshlack, 2010) was then applied on the gene counts, gene-expression values were log_2_-transformed, and observational-level theoretical variances were calculated using ‘voom’ for precision-weighting (Law et al., 2014).

### 2.8 Differential gene expression

To evaluate potential sources of overall gene expression variation, we performed Principal Component Analysis (PCA) on the *voom*-transformed gene expression (Hoffman and Roussos, 2020), plotting the samples along the first three principal components (Supplementary Fig. 3). To prepare the expression data for linear mixed effects modelling, we applied *voomWithDreamWeights* (‘variancePartition’ R package (Hoffman and Schadt, 2016) to the expression-level-filtered TMM-normalized counts. We then conducted linear mixed model regressions using *dream* (‘variancePartition’), which allows modelling of interindividual variability by adding individual identifiers as a random effect in the regression model, as shown below (Hoffman and Schadt, 2016, Hoffman and Roussos, 2020). We also included the fraction of all RNA sequencing ‘reads’ that were mapped to genes (i.e., ‘assigned percent’) for each sample as a covariate. Participant age was not included as a covariate on the assumption that reprogramming samples to stem cells negates age-related effects (Steg et al., 2021). All the participants were male, so gender was not included as a covariate. The final model with an interaction term between clinical group and treatment was as follows:

*Y_i_* = Group*Treatment + Assigned percent + Individual ID.

(Where “Group”, “Treatment” and “Assigned percent” were fixed effects and “Individual ID” was a random intercept effect).

Using contrasts, the following differential gene expression signatures were generated:

A. **Vehicle-treated SZ NPCs vs vehicle-treated control NPCs** (i.e., between SZ and control NPCs treated with vehicle).
B. **IFNγ-treated control NPCs vs vehicle-treated control NPCs** (i.e., the effect of IFNγ stimulation on gene expression in the control NPCs).
C. **IFNγ-treated SZ NPCs vs vehicle-treated SZ NPCs** (i.e., the effect of IFNγ stimulation on gene expression in the SZ NPCs).
D. **Interaction effect of IFNγ treatment in schizophrenia NPCs vs in control NPCs** (i.e., how the transcriptional response to IFNγ stimulation differs in SZ NPCs compared to control NPCs).
E. **IL-1β-treated control NPCs vs vehicle-treated control NPCs** (i.e., the effect of IL-1β stimulation on gene expression in the control NPCs).
F. **IL-1β-treated SZ NPCs vs vehicle-treated schizophrenia NPCs** (i.e., the effect of IL-1β stimulation on gene expression in the SZ NPCs).
G. **Interaction effect of IL-1β treatment in SZ NPCs vs in control NPCs** (i.e., how the transcriptional response to IL-1β stimulation differs in SZ NPCs compared to control NPCs).

Approximation of residual degrees of freedom and subsequent calculation of moderated eBayes *t*-statistics was done using the Satterthwaite method in ‘dream’ (Hoffman and Roussos, 2020).

### 2.9 Gene set enrichment analysis

Our gene set enrichment analyses (GSEA) included 935 unique gene sets: 519 immune-related and 421 nervous-system/neural function related (5 overlapping). Of these, 135 were obtained from previous literature (Pocklington et al., 2015, Pardiñas et al., 2018, Hall et al., 2020, Bhat et al., 2021) and the remaining from either the Molecular Signature Database (HALLMARK and Gene Ontology biological process gene sets) or the pathway databases KEGG, PANTHER, Pathway Commons and Reactome (see Supplementary Tables 4A-G for a full list of gene sets). GSEA assesses whether genes belonging to specific pathways or predefined sets of genes are over-represented in the significant or *peri*-significant results of a differential expression analysis. We used a linear mixed effects regression-based competitive gene set enrichment approach using the GSEA tool (Subramanian et al., 2005). GSEA was run on the seven signatures generated by the DGE analysis. We applied the *fgsea* function of the R package ‘fgsea’ (Korotkevich et al., 2019), using the standardized Z-score obtained in the differential expression analysis to rank the genes and running 100,000 permutations. All gene sets containing fewer than five genes were excluded. Multiple testing correction was performed within fgsea using the false discovery rate (FDR) method, and gene sets with an FDR < 0.05 were considered significant.

The resulting gene sets showed substantial constituent similarity (Supplemental Fig. 4), so we clustered them based on the overlap of the genes that belong to each gene set. This was done by calculating the Jaccard Similarity Index (which quantifies the intersection of two lists) between all pairs of significantly enriched gene sets and then applying a hierarchical clustering of gene sets based on the resulting dissimilarity matrix (1-Jaccard similarity). We then applied a cut-off of *h =* 0.5 to the dendrograms to obtain clusters of significantly enriched gene sets (Supplemental Fig. 5).

### 2.10 Enrichment of schizophrenia genes

To test whether differentially expressed genes in our experimental conditions were enriched for genes differentially expressed in *post-mortem* brain samples originating from SZ cases, we split genes by direction of effect (up- or downregulation) and assessed their overlap with genes differentially expressed in schizophrenia, according to Gandal et al. (2018). Significance of this overlap was estimated using the Fisher’s exact test in R, through the *GeneOverlap* package, assuming a genome size of 20,000 protein-coding genes.

To test whether the differentially expressed genes in our experiments were enriched for GWAS-supported genes, we performed gene-set enrichment analysis using MAGMA. The summary statistics file from the schizophrenia GWAS performed by Pardiñas et al. (2019) was downloaded and pre-processed using standard quality control procedures, where variants with minor allele frequency < 0.01, or those in the extended MHC region on chromosome 6, from 25 to 34 Mb, were removed. The GWAS variants were annotated to a list of protein-coding genes provided by the authors, which included genes located on chromosomes 1 to 22 and X, allowing a window of 35 kb upstream and 10 kb downstream of each gene, as described previously (de Leeuw et al., 2015). Gene-level enrichment analysis was performed to identify genes more likely to be associated with schizophrenia according to the GWAS results, using the European subset of the 1000 Genomes Phase 3 as reference panel. Subsequently, we tested whether there was an enrichment of genes differentially expressed in our experimental models (excluding those that were non-coding) within these results. All tests were corrected for multiple testing using the false discovery rate method, according to the number of gene sets analysed per condition (i.e., two gene lists [up- and downregulated genes] from two groups each [control vs. SZ cell lines] = 4 comparisons per analysis).

### 2.11 Validation by quantitative polymerase chain reaction (qPCR)

Reverse transcription of RNA to complementary DNA was carried out according to manufacturer’s instruction (SuperScript^TM^ III Reverse Transcriptase Invitrogen 18,080,093 and 40 U RNaseOUT Invitrogen 10777019). Forget-Me-Not™ EvaGreen® qPCR Master Mix (Biotium; 31041–1) was used for quantitative PCR on the QuantStudio 7 Flex Real-Time PCR System (Fisher) following the cycling parameters reported in Supplementary Table 3. Cycle threshold (Ct) values were normalized to the average of *GADPH, RPL13* and *SDHA* housekeeper Ct values.

## 3 Results

### 3.1 Demographic and sample details

The demographic and clinical characteristics of the six participants are described in Table 1. Subjects were male and of White British, or ‘Other White’ background. Ages ranged from 33 to 55 years old. The patients were diagnosed with paranoid schizophrenia, and controls were selected on the basis of having no history of neuropsychiatric disorders.

**Table 1:** Demographic and sample details.

**Cell line**	**Diagnosis**	**Year diagnosed**	**Medication**	**Age**	**Gender**	**Ethnicity**	**Reprogrammed by**
044	Schizophrenia	2011	Risperidone	33	Male	White British	Sendai virus
115	Schizophrenia	2010	Aripiprazol	43	Male	White British	Sendai virus
138	Schizophrenia	2008	Risperidone, Mirtazapine	39	Male	Black British	Sendai virus
M1	Control	–	–	55	Male	White British	Lentivirus
M2	Control	–	–	35	Male	White British	Lentivirus
M3	Control	–	–	35	Male	White British	Sendai virus

### 3.2 Validation of hiPSCs and NPCs

**Fig. 1:**
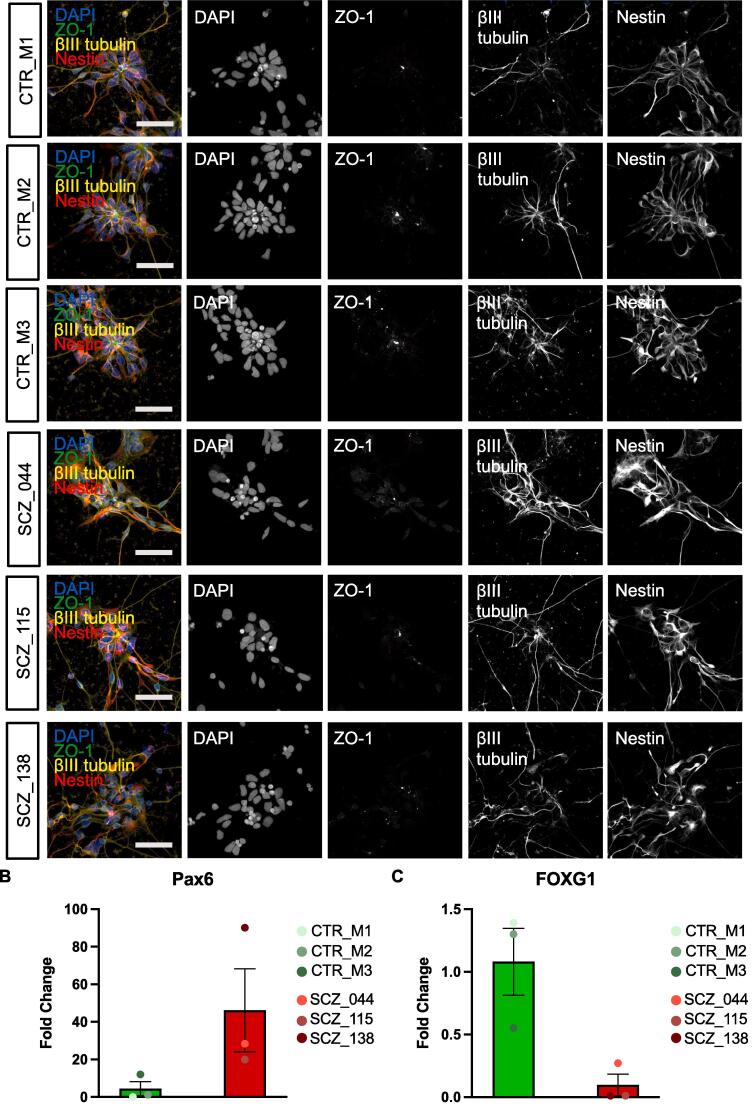
**Validation of neural progenitor cells. A.** Successful differentiation into neural progenitors was confirmed by staining at Day 20 for NPC markers, Nestin and β-III-tubulin. DAPI was used for baseline nuclear staining. Scale bar = 50 µm. **B.** Assessment of *PAX6* and *FOXG1* expression further supported the generation of NPCs following differentiation.

All hiPSC lines differentiated into embryoid bodies with characteristic three germ layers, and expression of pluripotency markers NANOG, OCT4, SSEA4 and TRA-1–81 (Supplementary Fig. 1A and B). Genome-wide SNP genotype data was used to derive schizophrenia polygenic risk score (PRS) using Psychiatric Genomics Consortium 3 genome wide association study (GWAS) summary statistics (Trubetskoy et al., 2022) for all hiPSC lines. This revealed that all SZ lines had a higher adjusted PRS compared to control lines (Supplemental Fig. 1C). For each participant, one clone was used for the NPC induction. All hiPSC lines successfully differentiated into NPCs as determined by immunostaining for known NPC markers βIII-tubulin and Nestin (Fig. 1A). Analysis of *PAX6* and *FOXG1* expression further supported that all hiPSC lines successfully differentiated into NPCs (Fig. 1B and C). We further assessed the expression of a range of NPC marker genes in the RNASeq data – this confirmed that that all hiPSCs were generating similar NPCs following differentiation (Supplementary Fig. 6).

### 3.3 Sources of variation in gene expression

We observed that the greatest source of variability across all samples was individual differences between the patients, as seen with principal component (PC) 1 (Supplementary Fig. 3 A-C, left panels) The importance of the clinical group was further supported by the clustering of SZ and control samples along the 2nd and 3rd principal components of PCA analysis (Supplementary Fig. 3C, right panel).

### 3.4 Differential expression of genes and gene set enrichment analysis (GSEA)

Of the seven comparisons we made, four showed significant differential gene expression at *FD*R < 0.05: the effect of diagnosis (Signature A), and the three IFNγ treatment conditions (Signatures B-D). There were no statistically significant (*FDR* 5%) differentially expressed genes (DEGs) as a result of acute IL1-β treatment. A full table of DEGs can be found in Supplementary Tables 3A-G. Immune-related gene sets were among the top ten most significantly enriched for all seven signatures, and synaptic transmission-related gene sets were among the top ten in four of the seven signatures. A full table of enrichment terms can be found in Supplementary Tables 4A-G. Details of DEGs and gene set enrichment analysis (GSEA) results for each signature are presented below.

### 3.5 Effect of schizophrenia diagnosis (Signature A)

**Fig. 2:**
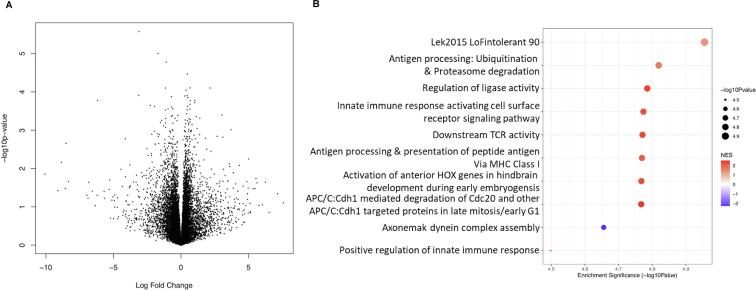
**Expression differences between NPCs from cases vs. controls (signature** **A) at the gene and pathway level. A.** The y-axis here shows statistical significance (-log10 p-value) of differential expression of genes in untreated cells from patient donors compared to gene expression in cells from untreated control donors. The x-axis shows the log_2_ fold change of expression of those genes in schizophrenia cell lines vs control cell lines. **B.** The top 10 significantly enriched gene set clusters (the gene set with the lowest p-value in each cluster is labelled on the x-axis). Data-points are sized according to significance (-log10 p-value) and coloured according to normalised enrichment score (NES), with blue indicating downregulation and red indicating upregulation. (For interpretation of the references to colour in this figure legend, the reader is referred to the web version of this article.)

We first investigated the gene expression differences observed in SZ relative to control lines, in vehicle-treated NPCs. We found only one statistically significant DEG (Fig. 2A), *AL132709.7*, a human-specific lncRNA gene which was overexpressed in patient lines (*FDR =* 0.0395; logFC = 3.111). Our sample was underpowered to detect other DEGs, but the GSEA revealed 26 significantly enriched gene-sets. The top five gene sets were enriched among nominally significant upregulated genes in this comparison (Fig. 2B), and the gene set with the lowest *p-*value was ‘Lek2015 loss-of-function (90)’ (*FDR* = 0.00098; normalized enrichment score (NES) = 1.36; genes in gene set = 3007), which contains 3007 genes that are intolerant to loss-of-function variants. This is consistent with previous schizophrenia genetic association studies that find associations with the same loss-of-function gene sets (Pardiñas et al., 2018, Hall et al., 2020).

### 3.6 Effect of IFNγ treatment in control NPCs (Signature B)

**Fig. 3:**
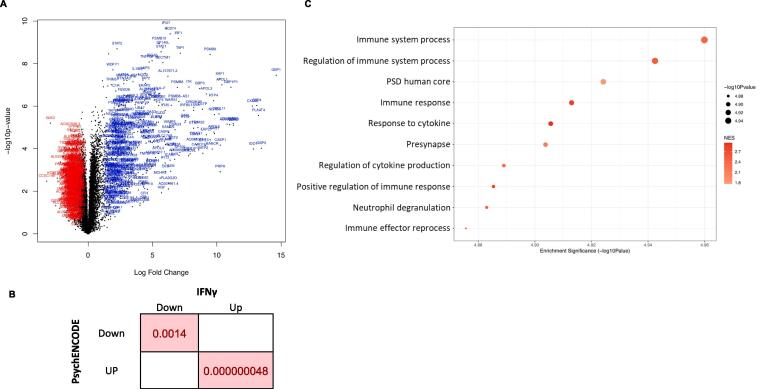
**Expression differences between NPCs from IFNγ treated versus untreated control NPCs (signature B) at the gene and pathway level. A.** The volcano plot shows, on the y-axis, the statistical significance (-log10 p-value) of differential expression of genes in IFNγ-treated control NPCs compared to untreated control NPCs. The x-axis is the magnitude of change (log_2_ fold change) in expression of those genes due to after IFNγ treatment. **B.** Enrichment (FDR) of SZ DEGs detected in the brains of patients with SZ within IFNγ-responding genes in treated control NPCs. Fisher’s exact test BH corrected for multiple comparisons. **C.** The top 10 significantly enriched gene set clusters (the gene set with the lowest *p*-value in each cluster is labelled on the y-axis). Please see Supplementary Spreadsheets 4A-G for full lists of enriched gene sets for each of the signatures. Data-points are sized according to significance (-log_10_ p-value) and coloured according to normalised enrichment score (NES), with blue indicating downregulation and red indicating upregulation. (For interpretation of the references to colour in this figure legend, the reader is referred to the web version of this article.)

We observed 1847 upregulated and 1533 downregulated genes in control NPCs (total = 3380 genes, out of 15,061 tested) in response to the acute IFNγ treatment, relative to the vehicle-treated lines (Fig. 3A and Supplementary Table 1). We observed significant upregulation of *STAT1* (*FDR =* 5.572 × 10^−6^; logFC *=* 5.680)*, STAT2* (*FDR =* 5.045 × 10^−6^; logFC *=* 2.247) and *JAK2* (*FDR =* 0.001; logFC = 2.111) – consistent with activation of the IFNγ signal transduction pathways) – as well as *IRF1* (*FDR =* 3.23 × 10^−6^; logFC *=* 7.022), a key downstream signalling target of this cytokine (Warre-Cornish et al., 2020). The genes whose expression was most significantly altered by IFNγ treatment were *IFI27* (*FDR* = 2.97 × 10^−6^; logFC = 6.067) and *CD274* (*FDR* = 2.97 × 10^−6^; logFC = 6.386), both upregulated. Three genes encoding guanylate-binding proteins were among those that showed the highest fold change (logFC): *GBP1* (*FDR* = 3.11 × 10^−5^; logFC = 14.622) *GBP5* (*FDR* = 0.0037; logFC = 13.473) and *GBP4* (*FDR* = 0.0002; logFC = 13.113). The upregulation of these genes is consistent with the role of guanylate-binding proteins (especially *GBP1*) in the inflammatory response associated with IFNγ (Honkala et al., 2019). Our findings for this comparison were also consistent with recent work which found MHC-I related genes among the most differentially expressed in IFNγ-treated control neural progenitors and neurons (Warre-Cornish et al., 2020). In our results (Supplementary Table  3A), key MHC-I related genes such as *HLA-A, HLA-B* and *HLA-C* are all consistently upregulated in response to IFNγ treatment.

We additionally tested whether the genes differentially expressed due to IFNγ treatment overlapped with genes known to be differentially expressed in patients with SZ, based on gene lists provided by (Gandal et al., 2018) (split by direction of effect: up- or down-regulation) and using the R package *GeneOverlap* (Shen, 2020). We observed a significant overlap between the genes downregulated in the IFNγ-treated control NPCs with genes downregulated in SZ (p = 7.00 × 10^−4^, *FDR* = 0.0014, odds ratio (OR) = 1.3), but not with those that were upregulated (p < 0.05) (Fig. 3B). Similarly, the genes upregulated in our model overlapped with those upregulated in SZ (p = 1.20 × 10^−8^, *FDR* = 4.80 × 10^−8^, OR = 1.5), but not with those that were downregulated (p < 0.05) (Fig. 3B). We performed another gene-set enrichment analysis, using MAGMA (de Leeuw et al., 2015), to test whether DEGs in our model were overrepresented in GWAS summary statistics from a large-scale schizophrenia GWAS (Pardiñas et al., 2018). This analysis did not identify evidence that IFNγ-regulated genes in our model were associated with interindividual genetic variation contributing to schizophrenia susceptibility (p < 0.05). Collectively, these results suggest that IFNγ signalling may impact neurodevelopment in a way that predisposes to schizophrenia, but that this may be independent from genetic effects. One caveat of the enrichment analysis using MAGMA is that it excludes genes within the MHC region due to the complex linkage disequilibrium structure at this locus, even though many of the genes in the MHC region are relevant for the IFNγ response.

We further observed 168 pathways were enriched when comparing IFNγ-treated cells and untreated cells in control NPCs. The gene set with the lowest *p-*value (Fig. 3C) was ‘immune system process’ from Gene Ontology (*FDR* = 0.0002; *NES* = 2.41; genes in gene set = 1235), which consists of genes involved in the development or functioning of the immune system. All of the top ten gene pathways for this signature were overexpressed amongst genes upregulated in response to IFNγ treatment. While most of these were related to the immune response initiated by cytokine exposure, we observed two that were, notably related to synaptic function: ‘post-synaptic density, human core’ and ‘presynapse’.

### 3.7 Effect of IFNγ treatment in schizophrenia NPCs (Signature C)

**Fig. 4:**
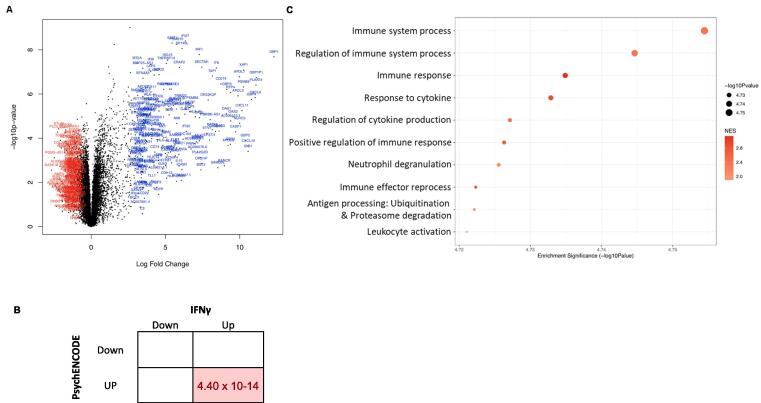
**Expression differences between NPCs from IFNγ treated versus untreated schizophrenia NPCs (signature** **C) at the gene and pathway level. A.** The volcano plot shows, on the y-axis, the statistical significance (-log_10_ p-value) of differential expression of genes in IFNγ-treated schizophrenia (SZ) NPCs compared to untreated SZ NPCs. The x-axis is the magnitude of change (log_2_ fold change) in expression of those genes due to after IFNγ treatment. **B.** Enrichment (FDR) of SZ DEGs detected in the brains of patients with SZ within IFNγ-responding genes in treated SZ NPCs. Fisher’s exact test BH corrected for multiple comparisons. **C.** The top 10 significantly enriched gene set clusters (the gene set with the lowest *p*-value in each cluster is labelled on the y-axis). Please see Supplementary Spreadsheets 4A-G for full lists of enriched gene sets for each of the signatures. Data-points are sized according to significance (-log_10_ p-value) and coloured according to normalised enrichment score (NES), with blue indicating downregulation and red indicating upregulation. (For interpretation of the references to colour in this figure legend, the reader is referred to the web version of this article.)

We observed 1061 upregulated and 919 downregulated genes in SZ cell lines (*FDR <* 0.05, total = 1980 genes, out of 15,061 tested) in response to the IFNγ treatment, relative to the vehicle-treated SZ lines (Fig. 4A and Supplementary Table 2). The genes whose expression was most significantly altered by IFNγ treatment in the SZ neural progenitors were *STAT2* (*FDR* = 1.46 × 10^−5^; logFC = 2.6103), *IFI27* (*FDR* = 1.74 × 10^−5^; logFC = 6.331) and *STAT1* (*FDR* = 1.74 × 10^−5^; logFC = 5.453). Once again, *IRF1* (*FDR* = 3.543 × 10^−5^; logFC = 7.278) and *JAK2* (*FDR* = 0.0033; logFC = 1.846) were also significantly upregulated. Here too, the highest logFC was shown by *GBP1* (*FDR* = 3.91 × 10^−5^; logFC = 12.308), followed by the pseudogene *GBP1P1* (*FDR* = 0.0001; logFC = 11.116).

We saw that genes upregulated in SZ NPCs treated with IFNγ (*FDR* < 0.05) also significantly overlapped with those known to be upregulated in SZ patients (p = 1.10 × 10^−14^, FDR = 4.40 × 10^−14^, odds ratio (OR) = 1.9) (but not those that were downregulated) (Fig. 4B). However, the downregulated genes in the model did not overlap with those downregulated (or upregulated) in cases (Fig. 4B), suggesting that the SZ NPCs may respond differently to IFNγ when compared to NPCs from unaffected individuals. Ultimately, we did not observe an enrichment of GWAS-supported variants within the genes differentially expressed in SZ NPCs after treatment, as observed in control NPCs (i.e., Signature B).

Nevertheless, there were 132 pathways enriched in the comparison of IFNγ-treated SZ cells (versus untreated SZ lines); the gene set with the lowest *p-*value (Fig. 4C) was, again, ‘immune system process’ (*FDR* = 0.0002; *NES* = 2.366; genes in gene set = 1235). The results in this comparison show activation of similar pathways in response to IFNγ in SZ lines as seen in control lines in the previous comparison (Fig. 4B). However, the transcriptional response appears attenuated, as we observed fewer DEGs overall.

### 3.8 Interaction effect of IFNγ treatment and schizophrenia diagnosis (Signature D)

**Fig. 5:**
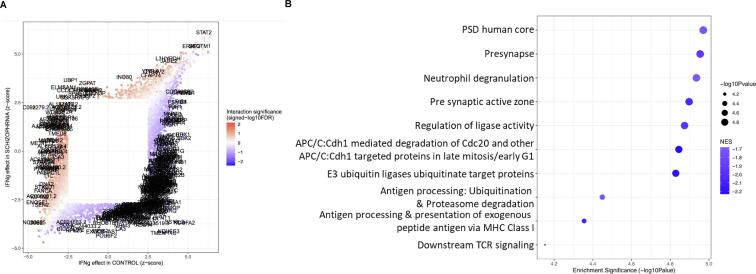
**Interaction effect between IFNγ-treatment and diagnostic group on gene expression (Signature D). A.** The scatterplot shows IFNγ response results for Signature in the 4137 genes that responded differentially to IFNγ in Signatures B and C. DEGs for control NPCs are on the *x*-axis and DEGs for schizophrenia NPCs are on the *y*-axis. The data are coloured by signed -log_10_FDR obtained for the interaction term (with blue indicating downregulation and red indicating upregulation). The 359 significant genes that are significant in the interaction are labelled. **B.** The top ten significantly enriched gene set clusters (the gene set with the lowest *p*-value in each cluster is labelled on the y-axis). Data-points are sized according to significance (-log_10_ p-value) and coloured according to normalised enrichment score (NES), with darker blue indicating greater downregulation. (For interpretation of the references to colour in this figure legend, the reader is referred to the web version of this article.)

To get a general picture of whether the patient NPCs respond differently to IFNγ treatment compared to how control NPCs do, we first examined the overlap of DEGs between signatures B and C (presented as a Venn diagram in Supplementary Fig. 7). It was evident that of the 4137 genes that responded to IFNγ in any of the two groups, only 1223 genes were in common to both, meaning that there are 2914 genes that appeared to respond differentially to IFNγ treatment between SZ and control NPCs. The following signature (Signature D) effectively assesses the same overlap; but subjects this comparison to an additional test of statistical significance (essentially omitting any of the 2914 genes observed in the initial comparison that may have differentially responded to treatment by chance). For this interaction term, we performed multiple-testing correction on the *p*-value obtained for the 4137 genes that are differentially expressed in response to IFNγ in any condition (controls and/or SZ cells). At *FDR* < 0.05 there were 359 genes that respond significantly differently to IFNγ between control and SZ cells (Fig. 5A; Table 2); most significantly the mitochondrial complex genes *NDUFA2* (*FDR* = 0.0003; logFC = -0.591) and *NDUFS3* (*FDR* = 0.0006; logFC = -0.330) and the lncRNA gene *AC092279.2* (*FDR* = 0.0006; logFC = 0.645). Indeed, the previous comparisons show that the mitochondrial genes are overexpressed in response to IFNγ in the control NPCs (logFC = 0.405, *NDUFA2*; logFC = 0.145, *NDUFS3*) but are underexpressed in response to IFNγ in SZ NPCs (logFC = -0.187*, NDUFA2*; logFC = -0.185, *NDUFS3*). *AC092279.2* shows the opposite profile, responding with underexpression in control cells (logFC = -0.517) and overexpression in SZ cells (logFC = 0.123).

**Table 2:** Top 20 genes significantly differentially expressed in IFNγ-treated compared to untreated cell lines in schizophrenia versus control NPCs (Signature D) – all downregulated. The right side of the table shows the effect of IFNγ treatment on the same genes in controls only, for comparison. See Supplementary Table 3D for differential expression results for all genes in this comparison. A negative logFC indicates downregulation.

	IFNγ effect in schizophrenia versus in control NPCs				IFNγ effect in control NPCs			
Gene Symbol	Log Fold Change	Average^2^ Expression	*p*-value	FDR^1^	Log Fold Change	Average Expression	*p*-value^3^	FDR^1^
*NDUFA2*	−0.591	4.976	1.00E-06	3.27E-04	0.405	4.976	0.000	0.000
*NDUFS3*	−0.330	6.229	5.00E-06	5.84E-04	0.145	6.229	0.000	0.005
*SS18L2*	−0.587	4.809	6.00E-06	5.84E-04	0.153	6.628	0.001	0.009
*TMEM14C*	−0.411	6.628	7.00E-06	5.84E-04	0.342	4.809	0.000	0.002
*AC092279.2*	0.645	3.906	9.00E-06	6.19E-04	−0.517	3.906	0.000	0.001
*BEX2*	−0.291	5.997	2.30E-05	1.38E-03	0.307	5.997	0.000	0.000
*RBX1*	−0.414	6.291	3.20E-05	1.64E-03	0.445	6.291	0.000	0.000
*MPLKIP*	−0.506	5.272	4.30E-05	1.93E-03	0.322	5.272	0.000	0.004
*COX6A1*	−0.605	5.983	4.90E-05	1.95E-03	0.418	5.983	0.000	0.003
*UQCRQ*	−0.669	5.772	5.50E-05	1.96E-03	0.392	5.772	0.000	0.006
*AL033519.3*	−1.604	0.605	6.90E-05	2.26E-03	0.651	0.605	0.004	0.026
*ALG14*	−0.597	3.048	1.29E-04	2.26E-03	0.176	3.048	0.024	0.082
*ATP5F1E*	−0.714	7.683	2.50E-04	2.26E-03	0.430	7.683	0.001	0.012
*BPNT1*	−0.661	4.865	8.50E-05	2.26E-03	0.307	4.865	0.001	0.012
*BTF3*	−0.371	8.866	1.91E-04	2.26E-03	0.168	8.866	0.003	0.025
*CA3*	−0.936	1.623	2.51E-04	2.26E-03	0.089	1.623	0.432	0.574
*CHCHD2*	−0.430	6.979	1.60E-04	2.26E-03	0.378	6.979	0.000	0.003
*COA3*	−0.507	5.485	2.22E-04	2.26E-03	0.490	5.485	0.000	0.002
*COPS9*	−0.804	5.617	9.30E-05	2.26E-03	0.512	5.617	0.000	0.007
*COX7C*	−0.552	7.975	1.50E-04	2.26E-03	0.409	7.975	0.000	0.005

1 False Discovery Rate.

2 Expression of the gene in (TMM-normalized) log_2_ CPMs (counts-per-million) averaged across all samples.

3 Uncorrected *p-*values.

**Table 3:** Top 20 gene sets significantly overrepresented among DEGs in IFNγ-treated compared to untreated cell lines in schizophrenia versus control NPCs (Signature D). The right side of the table shows the effect of IFNγ treatment on the same genes in control NPCs only, for comparison. Please see Supplementary Spreadsheet 4D for differential expression results for all genes in this comparison.

	IFNγ effect in schizophrenia versus in control NPCs					IFNγ effect in control NPCs		
Gene Set	Database	*p*-value	ES^1^	NES^2^	Number of genes in set	*p*-value	ES^1^	NES^2^
PSD human core	OP	1.07E-05	−0.32686	−1.719	654	1.19E-05	0.297	1.764
**Presynapse**	OP	1.11E-05	−0.37736	−1.944	465	1.25E-05	0.369	2.136
**Synaptic vesicle**	OP	1.15E-05	−0.35428	−1.787	353	1.30E-05	0.360	2.028
**Neutrophil degranulation**	Reactome	1.16E-05	−0.33523	−1.681	330	1.31E-05	0.444	2.483
**Presynaptic active zone**	OP	1.27E-05	−0.44027	−2.068	177	1.43E-05	0.402	2.085
**Regulation of ligase activity**	GO	1.34E-05	−0.44658	−1.994	121	1.53E-05	0.505	2.402
**Positive regulation of ligase activity**	GO	1.37E-05	−0.47269	−2.056	102	1.53E-05	0.505	2.402
**Cdc20:Phospho-APC/C mediated degradation of Cyclin A**	Reactome	1.43E-05	−0.54536	−2.224	70	1.58E-05	0.599	2.661
**APC/C:Cdh1 mediated degradation of Cdc20 and other APC/C:Cdh1 targeted proteins in late mitosis/early G1**	Reactome	1.43E-05	−0.54725	−2.232	70	1.58E-05	0.601	2.668
**APC/C:Cdc20 mediated degradation of Securin**	Reactome	1.44E-05	−0.57565	−2.316	65	1.60E-05	0.642	2.806
**Activation of NF-kappa-B in B cells**	Reactome	1.44E-05	−0.53276	−2.137	64	1.60E-05	0.648	2.826
**Autodegradation of Cdh1 by Cdh1:APC/C**	Reactome	1.45E-05	−0.57029	−2.281	63	1.60E-05	0.650	2.825
**E3 ubiquitin ligases ubiquitinate target proteins**	Reactome	1.48E-05	−0.57381	−2.195	50	1.64E-05	0.549	2.273
**Hedgehog ligand biogenesis**	Reactome	2.93E-05	−0.55385	−2.166	56	1.62E-05	0.684	2.900
**Cross-presentation of soluble exogenous antigens (endosomes)**	Reactome	3.01E-05	−0.58254	−2.170	44	1.65E-05	0.748	3.007
**Antigen processing: Ubiquitination & Proteasome degradation**	Reactome	3.56E-05	−0.34313	−1.697	283	1.34E-05	0.448	2.466
**Antigen processing and presentation of exogenous peptide antigen via MHC1**	GO	4.42E-05	−0.53871	−2.093	54	1.43E-05	0.549	2.833
**Downstream TCR signaling**	Reactome	7.04E-05	−0.4687	−1.958	80	1.57E-05	0.634	2.889
**Ligand gated channel activity**	GO	7.05E-05	0.40826	1.952	85	2.80E-05	−0.482	−2.412
**Reactive oxygen species pathway**	Hallmark	7.51E-05	−0.5478	−2.050	45	1.65E-05	0.579	2.338

There were 20 gene sets that were differentially expressed in this comparison. These gene sets were comprised of genes that showed different transcriptional responses to IFNγ in SZ NPCs compared to control NPCs (Fig. 5B). The most significantly different of these gene sets were ‘post-synaptic density (PSD), human core’ (*FDR* = 0.001; *NES* = -1.72; genes in gene set = 654), which includes several notable genes including the Alzheimer’s risk gene *APOE*, autism and schizophrenia risk genes *NRXN1, CYFIP1* and *SHANK1-3*, NMDA receptor gene *GRIN1,* and *DLG4,* which encodes the postsynaptic density protein PSD-95; as well as a ‘presynapse’ gene set, which includes genes that regulate the pre-synaptic ‘active zone’ and synaptic vesicle formation (Pardiñas et al., 2018, Pocklington et al., 2015; Pain et al., 2019) – notable genes in this gene set include *SV2A, MAOA* and several Na+/K + transport ATPase genes. In other words, genes influencing synaptic transmission showed a particularly attenuated response to IFNγ treatment in SZ NPCs.

### 3.9 Effect of IL-1β treatment on gene expression (Signature E, F & G)

**Fig. 6:**
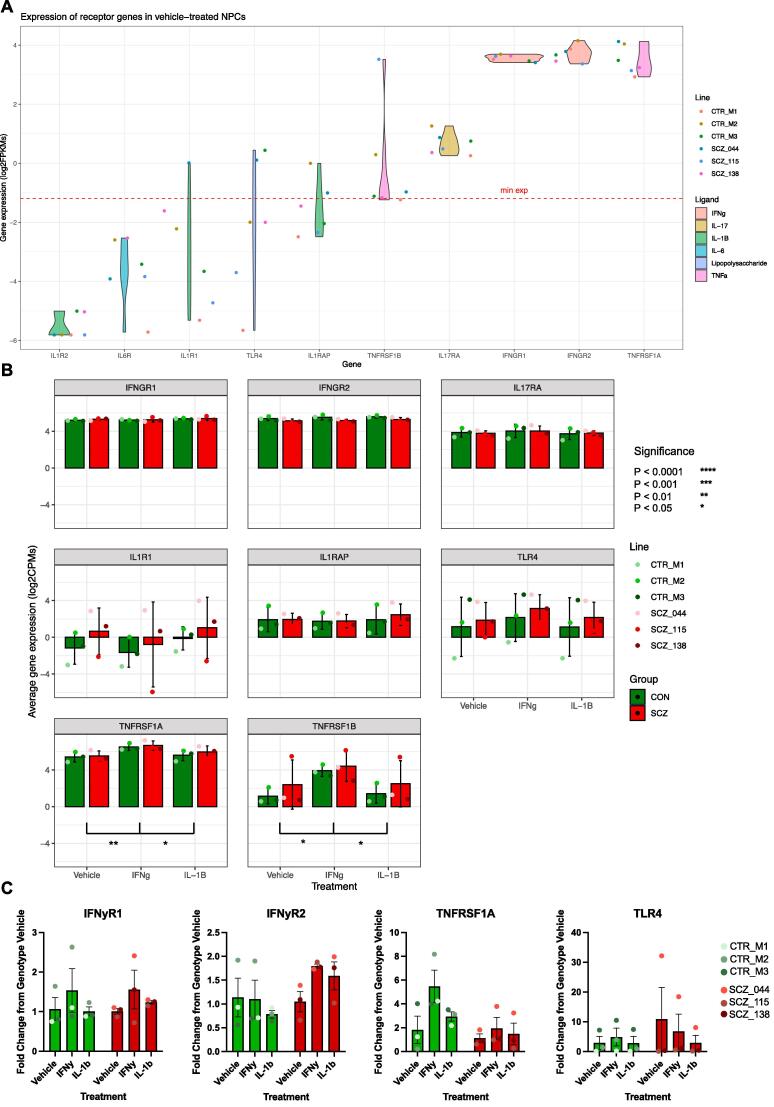
**Expression of cytokine receptors across all cell lines and conditions. A.** Distribution of cytokine receptor expression across all samples. Violin plots show expression (FPKMs) of cytokine receptor expression across all samples: Interleukin 1 Receptor 2 (*IL1R2*); Interleukin-6 Receptor (*IL6R*); Interleukin 1 Receptor 1 (*IL1R1*); Toll-Like Receptor 4 (*TLR4*); Interleukin 1 Receptor Accessory Protein (*IL1RAP*); TNF Receptor Superfamily Member 1B (*TNFRSF1B*); Interleukin-17 Receptor A (*IL-17RA*); Interferon Gamma Receptor 1 (*IFNGR1*); Interferon Gamma receptor 2 (*IFNGR2*); and TNF Receptor Superfamily Member 1A (*TNFRSF1A*). A minimum expression (‘min exp’) threshold (log_10_CPM = 0.6) is shown in red: IL1-beta receptor genes show negligible expression in NPCs. **B.** Effect of treatment on expression (log_2_CPMs) of cytokine receptor genes. The lower panels indicate a significant difference in expression of *TNFRSF1A* and *TNFRSF1B* with IFNγ treatment. C. QPCR validation of cytokine receptor expression across all samples and conditions. (For interpretation of the references to colour in this figure legend, the reader is referred to the web version of this article.)

We observed no differentially expressed genes associated with the effect of IL-1β treatment in either control (Signature E, Supplementary Figure 8A) or SZ (Signature F, Supplementary Figure 9A) NPCs (FDR > 0.05). We hypothesized that this could have been caused by the reduced expression of the main IL-1β receptors in cells at the neural progenitor stage. We assessed the expression of the genes encoding the IL-1β receptors *IL1R1, IL1R2,* and *IL1RAP,* and compared these to the expression of the IFNγ receptor genes *IFNGR1* and *IFNGR2*. We observed that the IL-1β receptor genes exhibited visibly lower expression relative to the IFNγ receptors (Fig. 6A and B), which may explain the lower responsiveness of the NPCs to IL-1β stimulation. In addition, these results suggest that IFNγ signalling may be more relevant than IL-1β at this developmental stage and/or for this cell type (NPCs).

**Fig. 7:**
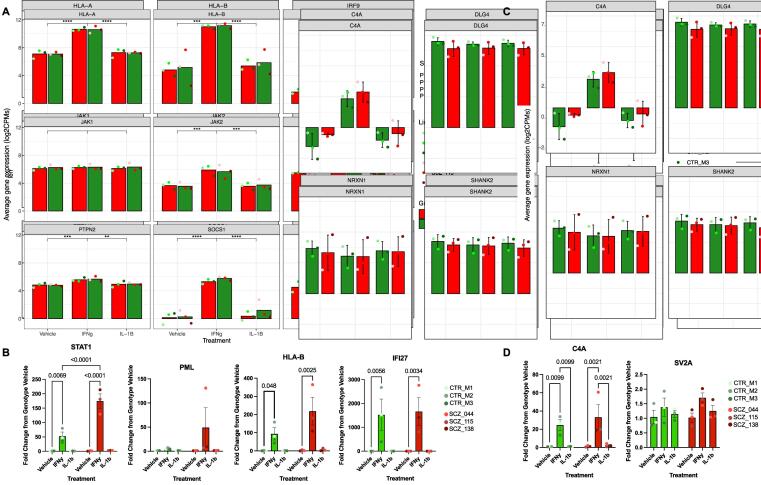
**Treatment-dependent expression of immune-response realted and synaptic gene in SZ and control NPCs. A.** Effect of treatment on expression (log_2_CPMs) of immune-response related genes. Treatment with IFNγ caused significant differences in expression in 8 out of 9 immune response-related genes, including genes related to MHC (*HLA-A, HLA-B, PML*) and negative regulators of *STAT1* signalling (*SOCS1, SOCS3* and *PTPN2*) in both control and SZ NPCs. **B.** Validation of IFNγ-induced changes in immune-response related genes in control and SZ NPCs by qPCR. This analysis further revelated a greater increase in *STAT1* expression in SZ NPCs compared to control cells following IFNγ treatment. **C.** Significant differences (p < 0.0001) were also observed in *C4A* expression as a result of IFNγ treatment in both SZ and control NPCs. **D.** This effect was further replicated in qPCR analysis of *C4A* expression.

Nevertheless, we observed 123 and 112 gene sets enriched for signatures E and F, respectively (Supplementary Figure 8B and 9B). For signature E, ‘regulation of immune system process’ was the most significant term (*FDR* = 0.0005; *NES* = 1.55; genes in gene set = 888); and ‘Lek2015 loss-of-function (90)’ (Pardiñas et al., 2018, Pocklington et al., 2015)(Pain et al., 2019) was the most significant term for signature F (*FDR* = 0.0008; *NES* = -1.97; genes in gene set = 3007). The fact that there are significant gene set enrichment terms for this comparison despite there being no DEGs suggests that there are indeed effects of IL-1β on transcription, but that our sample is underpowered to detect these individually. This method incorporates the expression signal from *peri*-significant genes and restricts the number of tests performed, reducing the multiple testing burden (instead of analysing 15,061 genes for differential expression, as in the DGE analysis, our enrichment analysis tests 895 gene sets).

As there were gene sets significantly enriched for these signatures, we also tested whether genes that were differentially expressed at a more lenient threshold of *FDR* < 0.1 were enriched for genes differentially expressed in SZ. We observed that genes upregulated in SZ NPCs treated with IL-1β showed a significant overlap with those upregulated in schizophrenia cases (p = 0.0013, FDR = 0.0052, odds ratio (OR) = 1.6). Further, using MAGMA, we observed that genes downregulated in the SZ NPCs were enriched with GWAS-supported variants associated with SZ (β = 0.19, SE = 0.06, P = 8.56 × 10^−4^, FDR = 3.42 × 10^−3^). While these results corroborate a role for IL-1β signalling in schizophrenia, it is likely that the effects of this cytokine on NPCs are limited due to the lack of other cell types (such as microglia) in the culture system used or occur at another this involves another developmental stage.

The interaction effect between IL-1β and SZ (Signature G) also did not yield any significantly differentially expressed genes (Supplementary Figure 10A) but did yield 15 gene sets that were significantly enriched. Of these, ‘regulation of ligase activity’ had the lowest *p-*value for signature G (*FDR* = 0.0015; *NES* = -2.15; genes in gene set = 121; Supplementary Figure 10B). These enrichment results also show a suppression of transcriptional response to IL-1β exposure in SZ NPCs, with pathways regulating the post-synaptic density and presynapse once again amongst the top ten (Supplementary Figure 10B).

### 3.10 Differential expression of key genes contributing to IFNγ effects

We further investigated the mechanisms that may have contributed to altered gene expression due to IFNγ treatment by conducting simple t-tests (with Benjamini-Höcheberg correction) on individual genes within our RNASeq data and by qPCR – in particular looking at key cytokine receptors (Fig. 6A-C), immune-related genes (Fig. 7A and B, Supplementary Figure 11A and B) and genes regulating synaptic transmission (Fig. 7C and Supplementary Figure 11C). Of the cytokine receptors analysed – Interleukin 1 Receptor 2 (*IL1R2*); Interleukin-6 Receptor *(IL6R)*; Interleukin 1 Receptor 1 (*IL1R1*); Toll-Like Receptor 4 (*TLR4)*; Interleukin 1 Receptor Accessory Protein (*IL1RAP*); TNF Receptor Superfamily Member 1B *(TNFRSF1B);* Interleukin-17 Receptor A *(IL-17RA)*; Interferon Gamma Receptor 1 (*IFNGR1*); Interferon Gamma receptor 2 (*IFNGR2*); and TNF Receptor Superfamily Member 1A *(TNFRSF1A)* – we saw a significant difference in expression of the TNFα receptor genes *TNFRSF1A* and *TNFRSF1B* between the IFNγ treatment condition and vehicle-treated and IL-1β treated cells. There was no significant difference for any of the receptor genes between SZ and control NPCs across treatment conditions.

Of the 9 immune response-related genes (*SOCS1, SOCS3* and *PTPN2,* which are negative regulators of *STAT1* signalling; *JAK1, JAK2,* which are key components of the IFNγ-dependent *JAK-STAT* pathway; and major histocompatibility complex (MHC) related genes, *HLA-A, HLA-B and PML*), significant differences (p < 0.01) were observed for all but *JAK1* as a result of IFN-gamma treatment (compared to both vehicle-treated and IL-1β treated cells).

Most interestingly, we saw significant differences (p < 0.0001) in *C4A* (complement component 4) gene expression as a result of IFNγ treatment (compared to both vehicle-treated and IL-1β treated cells). This is the case in both schizophrenia and control lines. Analysis by qPCR also confirms significant differences in expression of *IFI27* and *STAT1* in both SZ and control lines as a result of IFNγ treatment (Fig. 7B) – as seen in our DEG analysis (above). We also observe in our qPCR analysis that there is a significantly different (p < 0.0073) *STAT1* response to IFNγ treatment between patient and control lines (Fig. 7B).

## 4 Discussion

In this study, we sought to assess how the cytokines interferon-gamma (IFNγ) and interleukin-1 beta (IL-1β) interact with genetic profiles associated with schizophrenia (SZ), to better understand the increased susceptibility to schizophrenia seen in offspring of mothers exposed to infection during pregnancy. We hypothesised that cortical neural progenitor cells (NPCs) derived from patients with schizophrenia would respond differently to IFNγ or IL-1β exposure compared to those of healthy controls.

We performed a preliminary analysis to evaluate the transcriptomic differences between our SZ and controls NPCs without treatment stimulation and found only one differentially expressed gene. The identification of 26 gene sets significantly enriched for this comparison further supports the idea that, while there are noticeable differences between SZ and control NPCs at the transcriptomic level, our study may be underpowered to detect those for each gene individually.

We then assessed whether IFNγ treatment alters transcriptional responses in control NPCs and found that there were 3380 significant differentially expressed genes in response to IFNγ treatment. This is interesting itself, as the cell cultures used in this study do not contain glial cells – supporting the notion presented by our group in a recent study (Warre-Cornish et al., 2020) that human NPCs can launch an immune response independent of microglia, astrocytes or endothelial cells. Immune responses in the brain are thought to be predominantly mediated by glia (Greenhalgh et al., 2020), but the fact that NPCs are themselves responsive to a proliferation of IFNγ indicates that immunity in the brain extends beyond glial cells – as has recently been further evidenced by (Roy et al., 2022). This is also consistent with (Park et al., 2020), who show that neuronal co-culture with activated microglia is sufficient to induce deficits in the neurons – suggesting that cytokines might come from glia, but neurons can respond to them independently. Moreover, in both control and SZ NPCs, IFNγ treatment activated the canonical *JAK-STAT* signalling pathway, as would typically be seen in response to viral infection. Our findings for this comparison were also consistent with recent work which found MHC-I related genes among the most differentially expressed in IFNγ-treated control neural progenitors and neurons (Warre-Cornish et al., 2020). In our results (Supplementary Table 3A; Fig. 7A and B), key MHC-I related genes such *as HLA-A, HLA-B* and *HLA-C* are all consistently upregulated in response to IFNγ treatment. However, the genes most significantly upregulated on IFNγ exposure were *IFI27* and *CD274*. *IFI27* encodes Interferon Alpha Inducible Protein 27, which is involved in interferon-induced apoptosis and is considered to be a biomarker that differentiates between viral and bacterial infection (Tang et al., 2017). *CD274* encodes a receptor ligand that binds to PD-1 receptors on T-cell surfaces, inhibiting T-cell activation and antibody production – an essential process for preventing autoimmunity (Francisco et al., 2010). Interestingly, there is some evidence that *IFI27* is differentially expressed in transgenic mice that exhibit schizophrenia-like behaviours (Olaya et al., 2018). Similarly, *CD274* is a member of several gene-sets found to be enriched in a study of *de novo* copy number variant associated to SZ risk (Malhotra et al., 2011). In light of this evidence, these genes may be promising candidates for future studies exploring the link between MIA and SZ risk.

In SZ NPCs, there were fewer differentially expressed genes in response to IFNγ treatment: only 1980. We also observe in our qPCR results that there is a significantly different *STAT1* response to IFNγ treatment between patient and control lines. These findings may suggest that SZ cells are able to respond to IFNγ treatment, but overall maybe less able to activate a compensatory transcriptional response to infection. However, expression of other *JAK/STAT* and *HLA-* genes following IFNγ treatment did not significantly differ between SZ and control NPCs (Fig. 7; Supplementary Figure 11), indicating this difference may also be tied to intermediary variables unrelated to the IFNγ signalling pathway. For example, the two genes showing the most divergent response to IFNγ in SZ and control NPCs (*NDUFA2* and *NDUFS3*) were mitochondrial complex I genes, suggesting that schizophrenia donor cells are relatively driven to conserve energy in response to an infection, while healthy donor cells are able to expend more energy to restore health (Park et al., 2020). In support of this view, there is evidence for differences in the expression of mitochondrial genes in rodents susceptible to MIA (Mueller et al., 2021). This may also be related to pre-existing deficits in mitochondrial function in SZ lines, as it is well established that mitochondrial dysfunction contributes to the pathophysiology of SZ (Rajasekaran et al., 2015).

The gene sets significantly enriched for IFNγ treatment in both SZ and control NPCs largely converged in function, as expected, upon immune regulation. The gene sets that responded most differently to IFNγ in SZ lines were those regulating the postsynaptic density, presynapse, and presynaptic active zone. This result shows that the genes involved in the aberrant response to immune activation by SZ NPCs are involved in synaptic transmission, which fits with previous seminal work by Shatz (2009). These results are also consistent with additional models of SZ from other fields of neuroscience, including the concept of SZ as a disorder of synaptic ‘dysconnection’ in computational neuroscience – a promising bridge between two very different but equally rich views of the same disorder. The dysconnection hypothesis suggests a dysregulation of neuromodulation (particularly across glutamatergic synapses) lies at the core of the various factors contributing to SZ susceptibility (Adams et al., 2013; Friston et al., 2016).

Our results did not reveal any significant DEGs in response to IL-1β treatment in SZ or control NPCs. This is likely due to the low expression of the IL1 receptor 1 (*IL1R1*) gene that we observed, as blocking IL1R1 has previously been shown to significantly reduce the influence of IL-1β on NPCs (Crampton et al., 2012). However, this low IL-1 receptor expression was surprising in light of previous work showing high *IL1R1* (but not *IL1R2*) expression in rat ventral mesencephalon neural progenitors (Crampton et al., 2012). It is possible that there is lower *IL1R1* expression in the cortex than in other parts of the fetal brain; or perhaps *IL1R1* expression is upregulated at a later stage of prenatal neurodevelopment in humans than in rats, demonstrating the significance of leveraging human systems in the study of human neurodevelopment. It is also possible that that, by the 24-hour time point, any effect induced by IL-1β may have been lost – i.e., the effects of IL-1β may be very rapid and transient.

Nevertheless, pathway analyses did reveal significant enrichment of gene sets in response to IL-1β treatment, suggesting that there were transcriptional effects in both SZ and control NPCs in response to IL-1β, but our sample size only allowed their identification at the gene set level (at which the multiple-testing burden is smaller). The GSEA analyses revealed substantially different gene set enrichment profiles for the IL-1β signatures in SZ and control NPCs. Among the significantly enriched gene sets in control lines treated with IL-1β were (as with IFNγ) genes regulating the immune response, the presynapse and the post-synaptic density – all upregulated. However, none of these gene sets were among those most significantly enriched in IL-1β-treated SZ lines; instead, almost all of these were sets of genes involved in central nervous system development and neuronal morphogenesis – all downregulated. The genes that responded differently to IL-1β in SZ vs control NPCs were enriched in gene sets involved in cell division, antigen presentation and, once again, synaptic transmission. The most interesting finding to emerge from all these analyses was indeed that genes involved in synaptic transmission respond differentially to IFNγ and IL-1β exposure in schizophrenia NPCs compared to control NPCs.

In our RNAseq data, as well as our qPCR experiments, we observed significant differences in expression of complement component gene *C4A* – in both SZ and control cell lines – as a result of IFNγ treatment. IFNγ has been previously reported to regulate C4A synthesis (Kulics et al., 1990). As Sekar et al. (2016) established in their seminal study, *C4A* is a key risk factor for schizophrenia and may confer this susceptibility by stimulating excessive synaptic pruning by microglial phagocytosis. This is further supported by Sellgren et al. (2019)), who showed, using in vitro microglial-neuronal cultures derived from human cells, that SZ cells exhibit increased synaptic elimination by microglia. This finding further implicates the influence of IFNγ on synaptic development in developing neurons. We also observe significant overexpression of *JAK2, STAT1, IRF1* and TNFα receptor genes as a result of IFNγ treatment, which is in line with previous evidence that TNFα and IFNγ co-regulate the *JAK/STAT1/IRF1* pathway (Karki et al., 2021).

The current study was primarily limited by the relatively small sample size, which would warrant future replication studies. It was also surprising to find that donor age had some influence over the variance in gene expression in the sample, as one would also expect a negation of age-related epigenetic effects (Steg et al., 2021). It is likely that this is due to noise (again as a result of the small sample size), which emphasises the importance of replicating these findings. We also administered only a single, acute (24 hour) dose of cytokine treatment to our NPC cultures: in future studies, it would be interesting to examine the effect of chronic treatment. For IL-1β effects, future studies looking at different developmental time points and cell types could elucidate this cytokine’s role in neurodevelopmental processes associated with schizophrenia. Furthermore, our findings suggest that cortical NPCs are more responsive to IFNγ than IL-1β. Whether this is the case in other cell types such as astrocytes and microglia would be an interesting area for further investigation, especially in SZ patient cell lines. As yet, there have only been a few studies with findings that are relevant to this question in induced cell lines from patients with SZ. In induced microglia from patients with SZ, (Ormel et al., 2020) see an increase in TNFα secretion in response to lipopolysaccharide (LPS). In hiPSC-derived astrocytes, there is evidence for effects of IL-1β that may differ by diagnosis (Akkouh et al., 2020).

In summary, having conducted the first transient cytokine exposure study using hiPSC-derived NPCs from patients with SZ, we have found that immune activation induced by IL-1β and IFNγ elicits transcriptional changes that may alter the course of subsequent neurodevelopment. There were two particularly significant take-home messages from this study, as follows. First, there does appear to be a significant transcriptional response to IFNγ treatment in NPCs, with differential expression implicating mitochondrial complex genes, which are underexpressed in response to treatment in SZ lines. Second, our findings highlight pre- and post-synaptic genes as differentially expressed in response to IFNγ, and differentially regulated in response to treatment in SZ NPCs. In other words, SZ NPCs do not upregulate synaptic genes in response to a cytokine challenge as much as control NPCs do. This is also consistent with previous literature, including large-scale transcriptome-wide association studies (Hall et al., 2020) and the recent Psychiatric Genetics Consortium study showing synaptic genes to be the most enriched for schizophrenia risk (Trubetskoy et al., 2022; Ripke et al., 2019). This is particularly interesting as NPCs do not have synapses. It could be that these early changes impact synaptic development after these cells differentiate into neurons. Indeed, our previous research shows that IFNγ induces molecular and cellular changes in NPCs that persist even when these cells differentiate into neurons (Warre-Cornish et al., 2020). Our findings exemplify differences in how the brains of people with SZ may have responded to infection or inflammation during prenatal development and suggest immune insults early in life can alter neurotransmission. Finally, we identify new gene targets for future research on the influence of maternal immune activation on SZ susceptibility and resilience.

## Author contributions

A.B. wrote the manuscript, conducted the cell culture and experimental treatments, and worked with H.I. on quality control of the genetic data and statistical analyses. H.I. worked on the analyses as well as imputation and quality control of the genetic data and edited the manuscript. P.R., L.D.P and R.N. assisted with the cell culture and experimental treatments. R.R.D conducted the *GeneOverlap* analyses and edited the manuscript. A.C.V., T.P, E.B. and J.P. edited and provided guidance on the manuscript and interpretation of the results. G.M., C.I., P.J.M.D., C.S. and R.N. set up the experimental pipelines for the lab in which the experiments were conducted and were involved in sample collection and reprogramming of keratinocyte samples. A.C. conducted the qPCR experiments under the supervision of A.C.V. and B.H. calculated polygenic risk scores. S.B. was responsible for recruitment and clinical evaluation of patients whose hair samples were used in this study. D.P.S is the principal investigator, corresponding author and PhD supervisor to A.B., and provided essential guidance throughout, as well as editing the manuscript and figures. A.B., A.C.V. and D.P.S. conceived the project and were responsible for the design of experiments. All of the co-authors provided intellectual input to the study.

## Declaration of Competing Interest

The authors declare that they have no known competing financial interests or personal relationships that could have appeared to influence the work reported in this paper.
