# Supplementary material for: Attenuated transcriptional response to pro-inflammatory cytokines in schizophrenia hiPSC-derived neural progenitor cells
Source: Brain Behav Immun. 2022 Oct;105:82–97. doi: 10.1016/j.bbi.2022.06.010 (PMC9810540; doi:10.1016/j.bbi.2022.06.010)
Supplement: Supplementary data 1 [file mmc1.pdf]

Attenuated transcriptional response to pro-inflammatory cytokines in schizophrenia hiPSC-derived neural progenitor cells

Anjali Bhat[1,2,3,4], Haritz Irizar[3,8], Amalie Couch[1,2], Pooja Raval[1,2], Rodrigo R.R. Duarte[5,9], Lucia Dutan Polit[1,2], Bjorn Hanger[1,2], Timothy Powell[5,9], P.J. Michael Deans[1,2], Carol Shum[1,2], Roland Nagy[1,2], Grainne McAlonan[2,6], Conrad O. Iyegbe[7], Jack Price[1,2], Elvira Bramon[3, 7], Sagnik Bhattacharyya[8], Anthony C. Vernon[1,2§*], Deepak P. Srivastava[1,2§*]

Supplementary information, tables and figures.

Supplementary Tables 1, 2 & 5

Supplementary Figures 1 - 11

Supplementary Methods

Calculation of Schizophrenia Polygenic Risk Scores

Schizophrenia polygenic risk scores (PRS) were calculated for all six lines used in the current study, to validate the grouping of patient and control lines (Supplementary Figure 11). Output files from the Infinium PsychArray-24 Kit (Illumina) were imported to Illumina GenomeStudio (v 2.0 .5) and low-quality SNPs were filtered out with recommended settings. Data was then exported to PLINK [1] format using the GenomeStudio associated plugin (PLINK Input Report Plug-in v2.1.4) on which QC was performed. With PLINK, variants with < 99 % call rate, minor allele frequency (MAF) < 1 %, missing genotype rate < 1%, and that were outside of the Hardy-Weinberg equilibrium (p < 10-5) were excluded. Due to low sample size, linkage disequilibrium was estimated from non-Finnish European participants in the 1000 Genomes study [2]. Imputation was performed using the Sanger Imputation Service to the Haplotype Reference Consortium panel using the EAGLE2+PBWT panel [3, 4]. In PLINK, the same QC was repeated after merging files. To generate PRS, the PRSice2 software [5] was used with default options, using the –all-score output to designate individual PRS for each cell line at the different *p*-value thresholds (P(T) 0.001, 0.05, 0,1, 0,2, 0,3, 0,4, 0,5 and 1). The *p*-value threshold that explained the largest amount of variance (p 0.001) was used as designated PRS. The Psychiatric Genomics Consortium (PGC2) schizophrenia meta-analysis [6] was used as the training sample with the imputed Psycharray files as the target.

Supplementary Tables

Supplementary Table 1. Top 20 genes most significantly differentially expressed in IFNγ-treated control cells compared to untreated control cells.

Gene Symbol	Log fold change	Average expression[2]	P value	Adjusted P value	Z score	FDR[1]
IFI27	6.067	4.280	2.16E-10	2.97E-06	6.350	2.97E-06
CD274	6.386	1.547	3.95E-10	2.97E-06	6.256	2.97E-06
IRF1	7.022	4.265	6.44E-10	3.24E-06	6.179	3.23E-06
PSMB10	5.522	1.528	1.15E-09	4.32E-06	6.087	4.33E-06
SP140L	5.815	0.465	1.81E-09	5.05E-06	6.014	5.05E-06
STAT2	2.247	6.869	2.01E-09	5.05E-06	5.997	5.05E-06
STAT1	5.680	7.882	2.74E-09	5.58E-06	5.946	5.57E-06
TAP1	7.167	4.928	3.18E-09	5.58E-06	5.922	5.57E-06
GSTK1	1.608	5.890	3.47E-09	5.58E-06	5.908	5.57E-06
PSMB9	9.482	2.362	3.70E-09	5.58E-06	5.897	5.57E-06
ISG15	4.980	4.931	7.22E-09	9.89E-06	5.786	9.88E-06
TNFRSF14	4.751	1.658	8.30E-09	1.04E-05	5.762	1.04E-05
SECTM1	5.813	0.609	9.07E-09	1.05E-05	5.747	1.05E-05
WDFY1	1.922	7.012	1.94E-08	2.09E-05	5.617	2.09E-05
LAP3	4.433	6.512	2.85E-08	2.86E-05	5.551	2.86E-05
IL18BP	3.892	4.188	3.13E-08	2.95E-05	5.534	2.95E-05
GBP1	14.622	2.693	3.51E-08	3.11E-05	5.514	3.11E-05
AL157871.2	6.200	-0.603	3.88E-08	3.25E-05	5.496	3.25E-05
XAF1	10.252	-1.583	5.23E-08	4.15E-05	5.443	4.15E-05

[1] False Discovery Rate

[2] Average expression of the gene in TMM-normalised $\log_2$ CPMs (counts-per-million).

(Please see Supplementary Spreadsheets 3A-G for all genes included in the differential expression comparisons.)

Supplementary Table 2. Top 20 genes most significantly differentially expressed in IFNγ-treated schizophrenia cell lines compared to untreated schizophrenia cell lines.

Gene Symbol	Log fold change	Average expression[2]	P value	Adjusted P value	Z score	FDR[1]
STAT2	2.610	6.869	9.67E-10	1.46E-05	6.115	1.46E-05
IFI27	6.333	4.280	4.00E-09	1.74E-05	5.884	1.74E-05
STAT1	5.454	7.882	4.90E-09	1.74E-05	5.851	1.74E-05
PSMB10	5.740	1.528	5.49E-09	1.74E-05	5.832	1.74E-05
GSTK1	1.552	5.890	5.77E-09	1.74E-05	5.823	1.74E-05
SP140L	6.125	0.465	8.51E-09	2.14E-05	5.758	2.14E-05
IRF1	7.278	4.265	1.65E-08	3.54E-05	5.646	3.54E-05
GBP1	12.308	2.693	2.08E-08	3.91E-05	5.606	3.91E-05
ISG15	5.142	4.931	2.92E-08	4.88E-05	5.546	4.88E-05
MT2A	3.091	4.452	3.97E-08	5.57E-05	5.492	5.57E-05
TNFRSF14	5.052	1.658	4.07E-08	5.57E-05	5.488	5.57E-05
IFI6	4.015	6.238	4.57E-08	5.73E-05	5.467	5.73E-05
ADAR	1.182	8.246	5.86E-08	6.02E-05	5.423	6.02E-05
SECTM1	7.449	0.609	6.01E-08	6.02E-05	5.419	6.02E-05
ERAP2	5.936	2.491	6.23E-08	6.02E-05	5.412	6.02E-05
ITK	8.431	-1.891	6.66E-08	6.02E-05	5.400	6.02E-05
MMP25-AS1	3.487	2.799	6.80E-08	6.02E-05	5.396	6.02E-05
XAF1	10.277	-1.583	8.11E-08	6.79E-05	5.365	6.79E-05
LAP3	4.017	6.512	9.33E-08	7.40E-05	5.339	7.40E-05

[1] False Discovery Rate

[2] Average expression of the gene in TMM-normalised $\log_2$ CPMs (counts-per-million).

<table>
<tr><th>Step</th><th>Temperature (ºC)</th><th>Time</th><th>Cycle</th></tr>
<tr><td>Initial denaturation</td><td>95</td><td>10 mins</td><td>1</td></tr>
<tr><td>Denaturation</td><td>95</td><td>15 sec</td><td rowspan="3">40</td></tr>
<tr><td>Annealing</td><td>60</td><td>30 sec</td></tr>
<tr><td>Extension</td><td>72</td><td>30 sec</td></tr>
</table>

Supplementary Table 5. Cycling parameters used during qPCR.

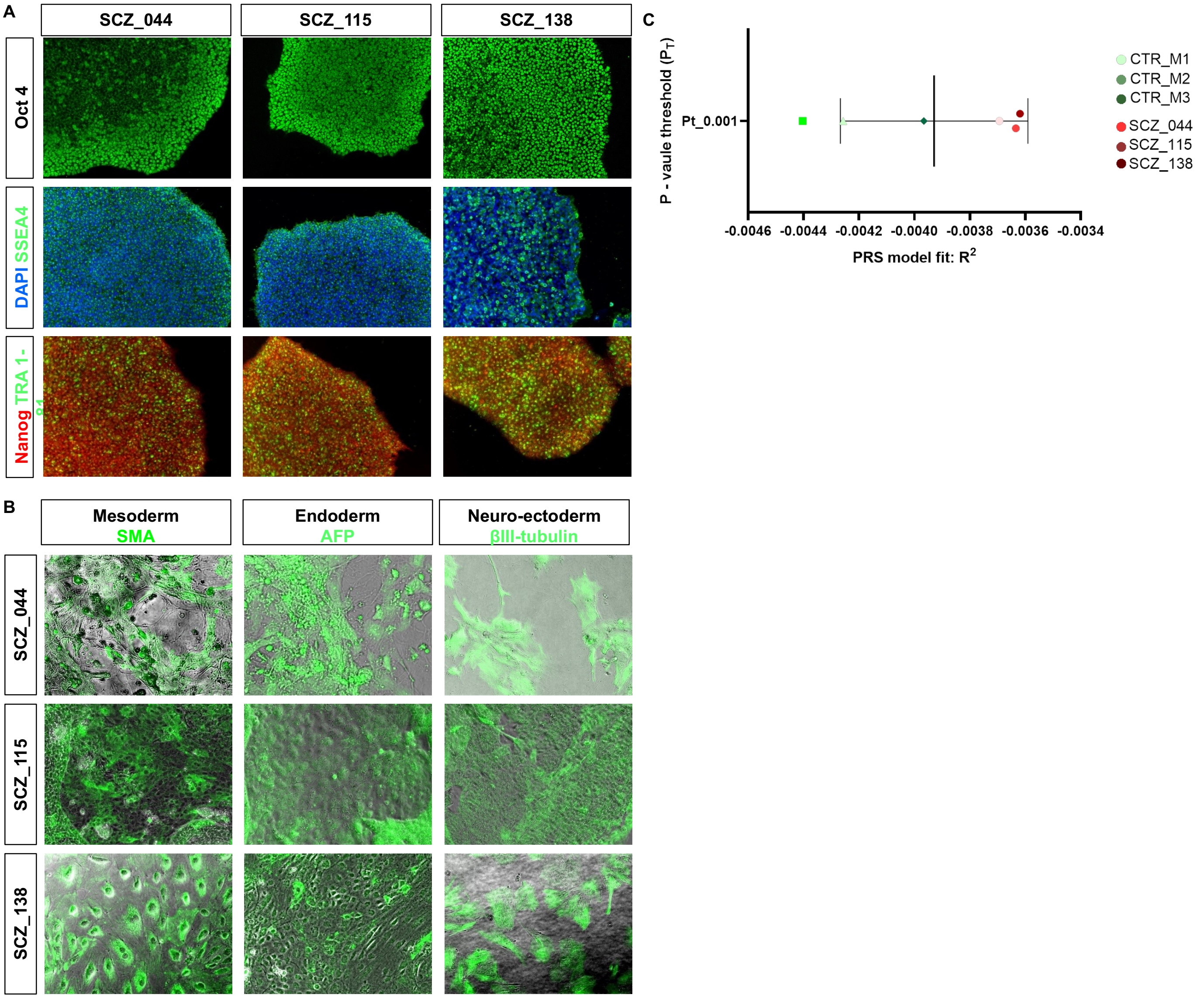

Supplementary Figure 1. Validation of schizophrenia hiPSC lines. A. Immunostaining of iPSCs for the pluripotency markers OCT4, SSEA4, NANOG and TRA-1-81. **B.** SZ hiPSCs generated 3 germ layers - hiPSC lines showed spontaneous generation of mesoderm, endoderm and neuro-ectoderm cells. Validation of control lines used in this study has been shown in [32, 33]. **C.** Schizophrenia Polygenic Risk Scores (PRS) for each cell line, confirming our grouping of SZ and control lines in terms of genetic risk: SZ lines show a higher PRS than control lines. Patient and control lines cluster away from each other and are not widely separated within group, indicating that there is no variable penetrance of schizophrenia risk genes.

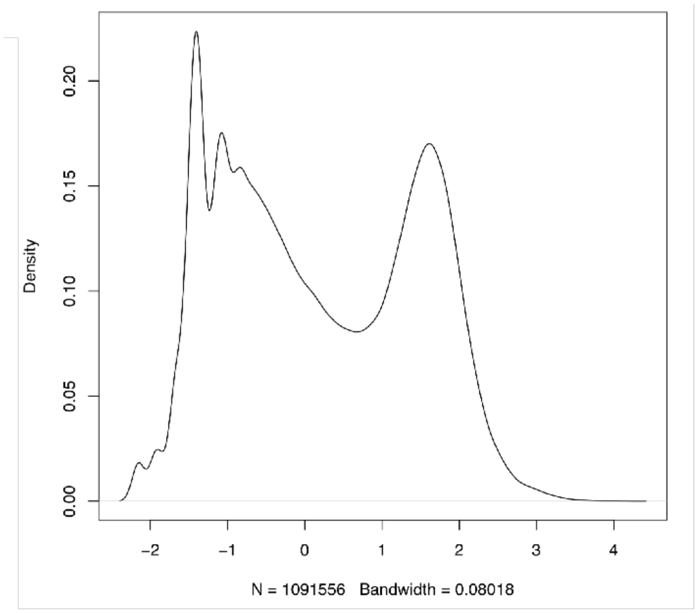

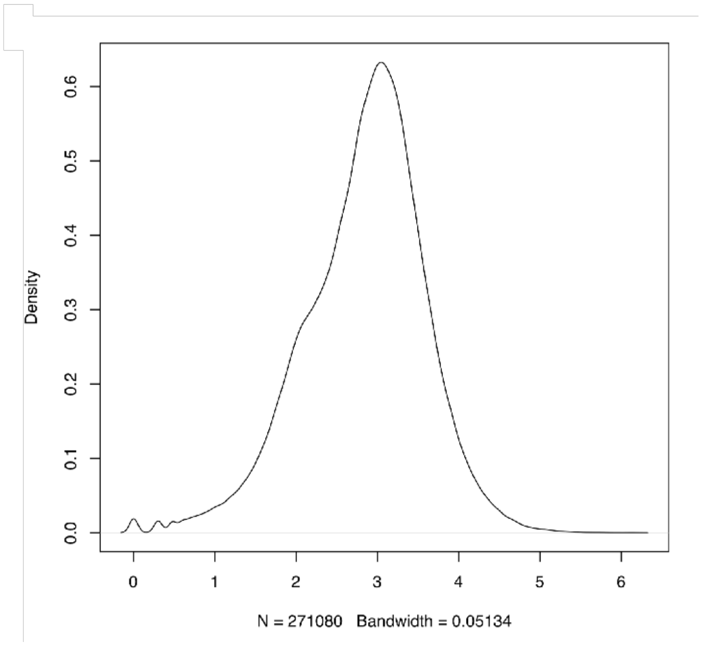

Supplementary Figure 2. *Filtration to remove lowly expressed genes in order to minimise technical noise.* Plots **A** and **B** show the distribution of log_{10}-transformed counts-per-million (CPMs) pre-filtration and post-filtration, respectively. Filtration threshold was set at $log_{10}CPM = 0.6$ (CPM x $10^{0.6}$) by visual inspection, such that the filtered data showed an approximate Gaussian distribution.

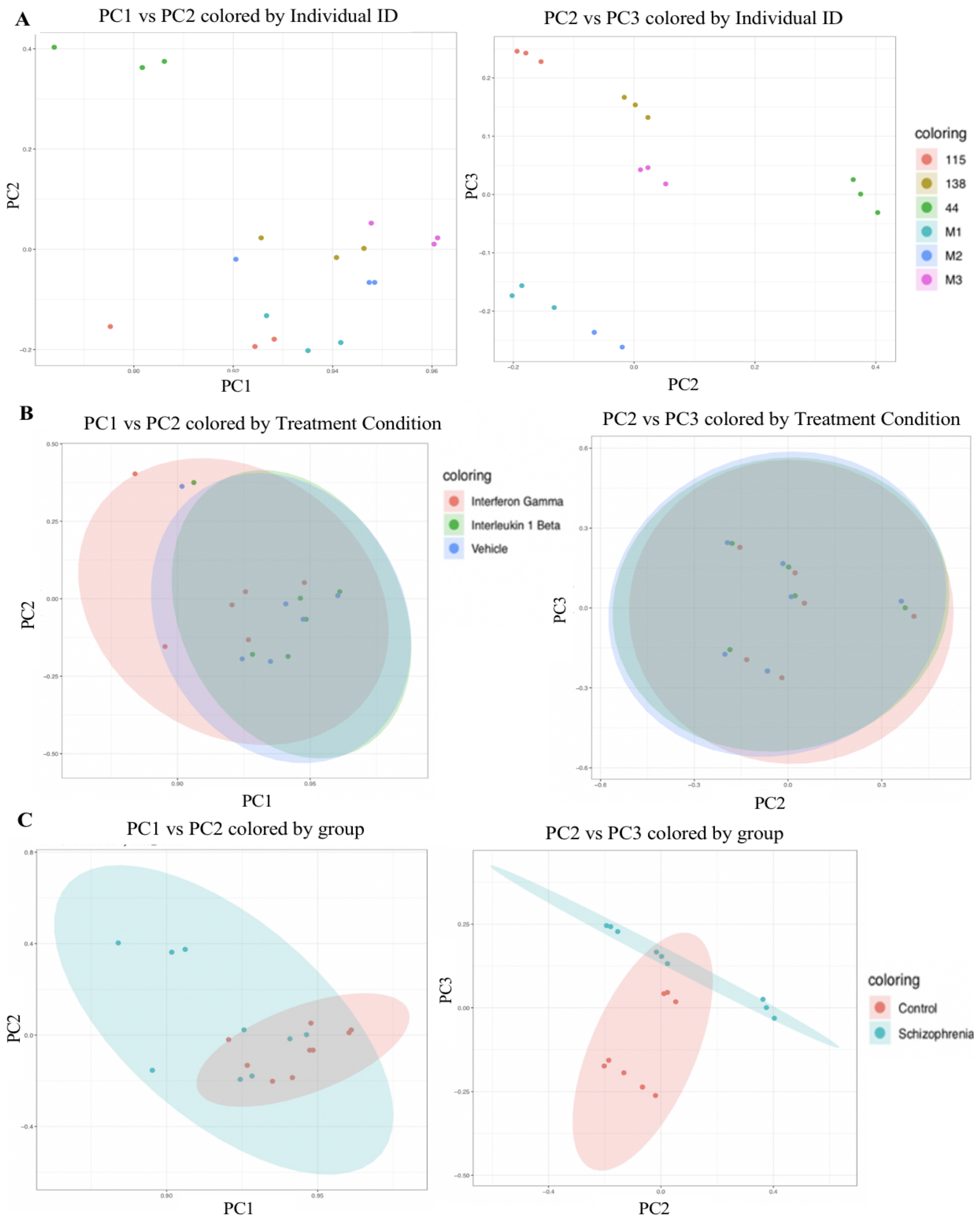

Supplementary Figure 3. *Principal component analysis (PCA).* The left panels show the relationship between PC1 and PC2; the right shows PC2 against PC3 (PC1 accounts for 86.55% of the variation in the data). **A.** By individual identifier. **B.** By treatment condition: IFNγ, IL-1β and vehicle (untreated). **C.** By diagnostic group (schizophrenia versus controls).

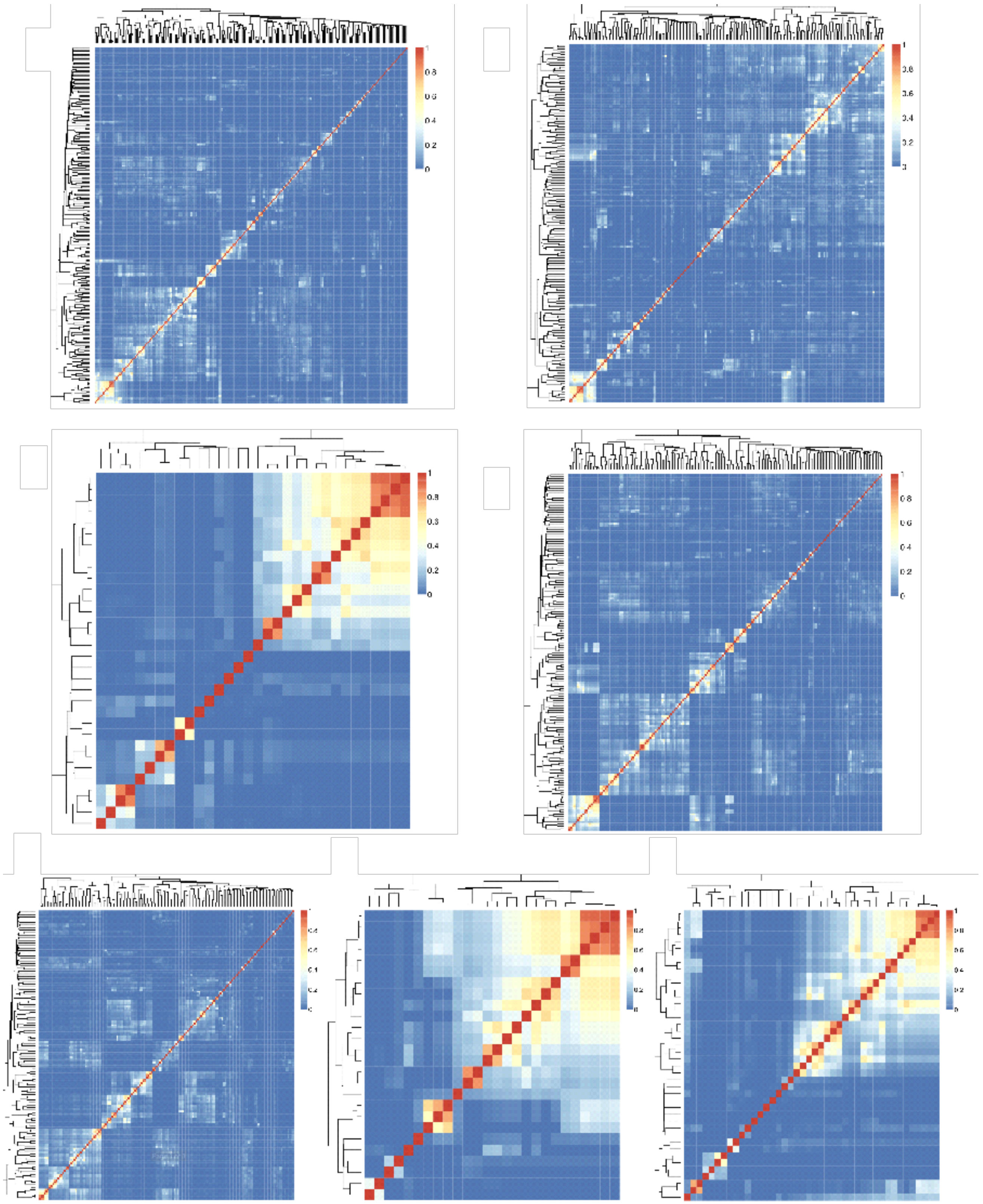

Supplementary Figure 4. *Jaccard similarity matrices* for signatures A-G, respectively

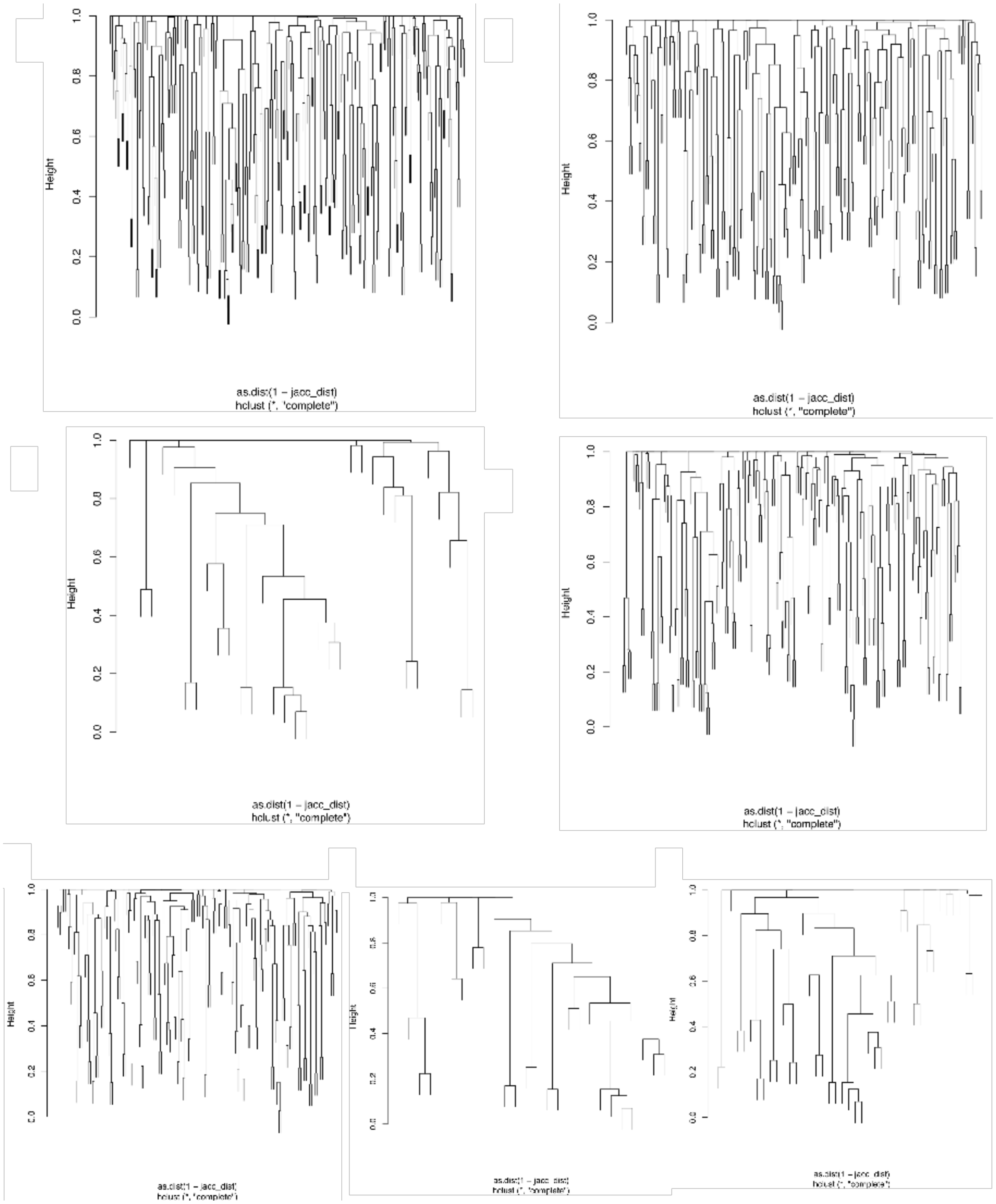

Supplementary Figure 5. *Cluster dendograms* for signatures A-G, respectively.

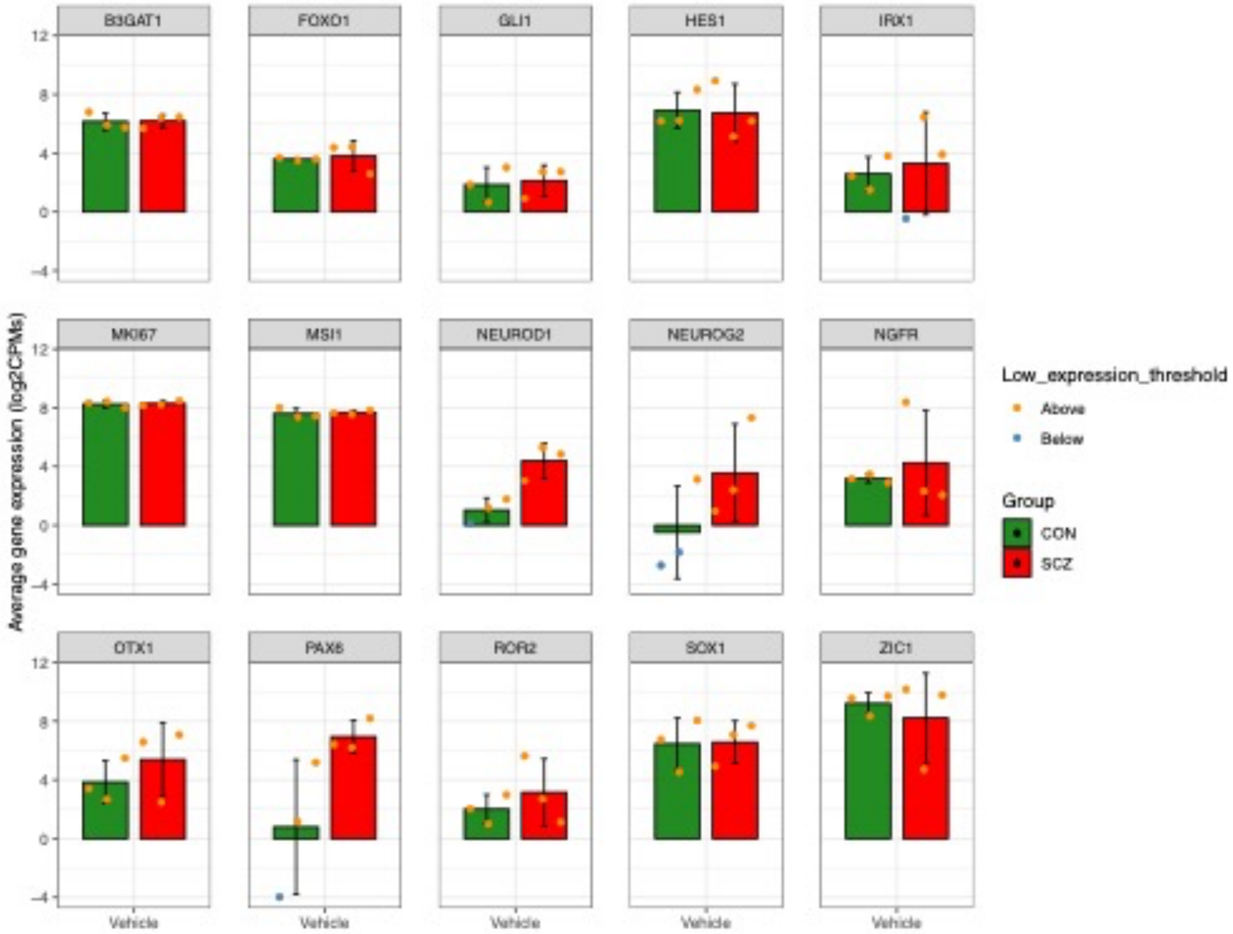

Supplementary Figure 6. Expression of NPC markers (log_2 CPMs) in vehicle-treated schizophrenia (red) and vehicle-treated control (green) hiPSC lines. No difference in expression is seen indicating the generation of a similar population of NPCs following differenation.

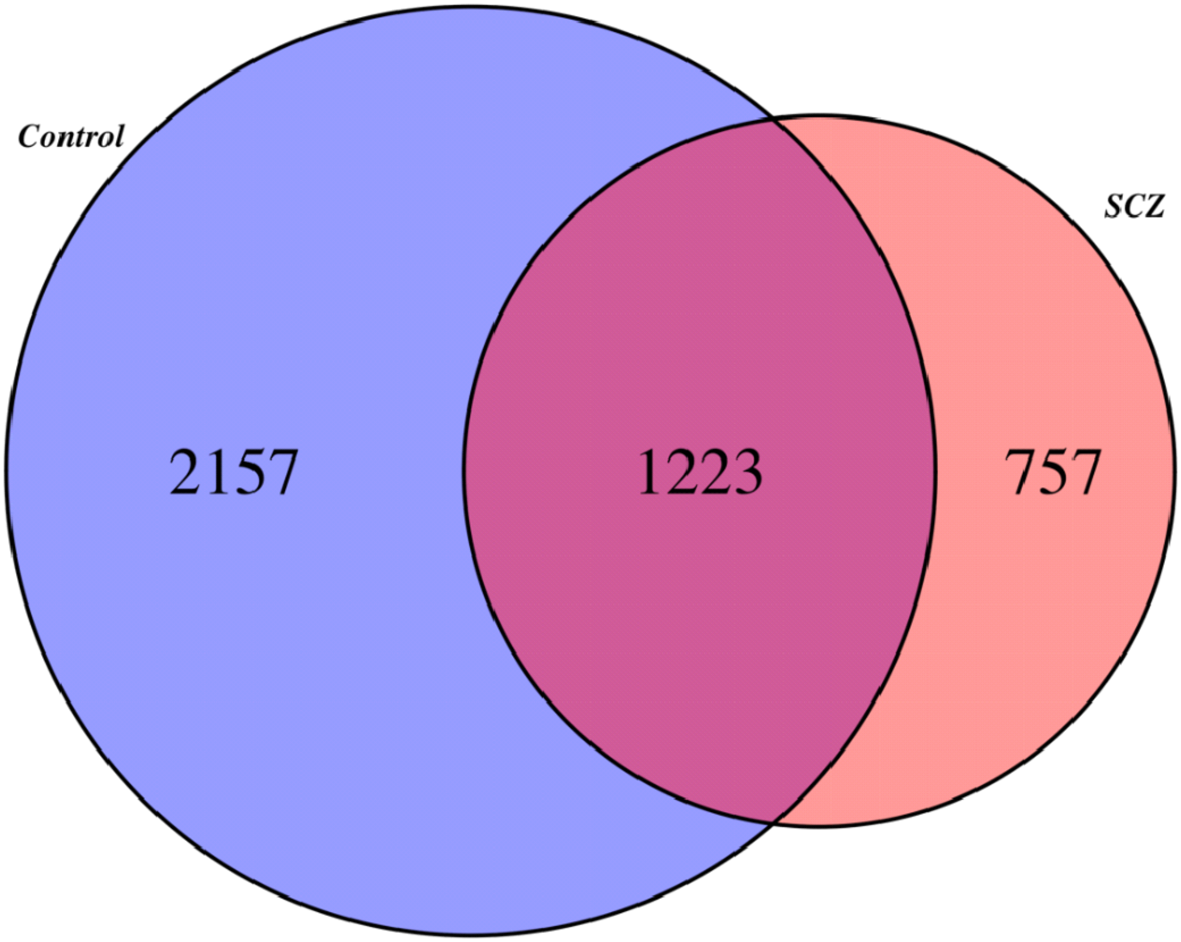

Supplementary Figure 7. *Venn diagram that shows the overlap between the genes that are differentially expressed in response to IFNγ in control cells and in schizophrenia cells.* Of the 4137 genes that respond to IFNγ in any of the two groups, only 1223 genes are in common, meaning that there are 2914 genes that appear differentially expressed in response to IFNγ in controls vs not in schizophrenia and vice versa.

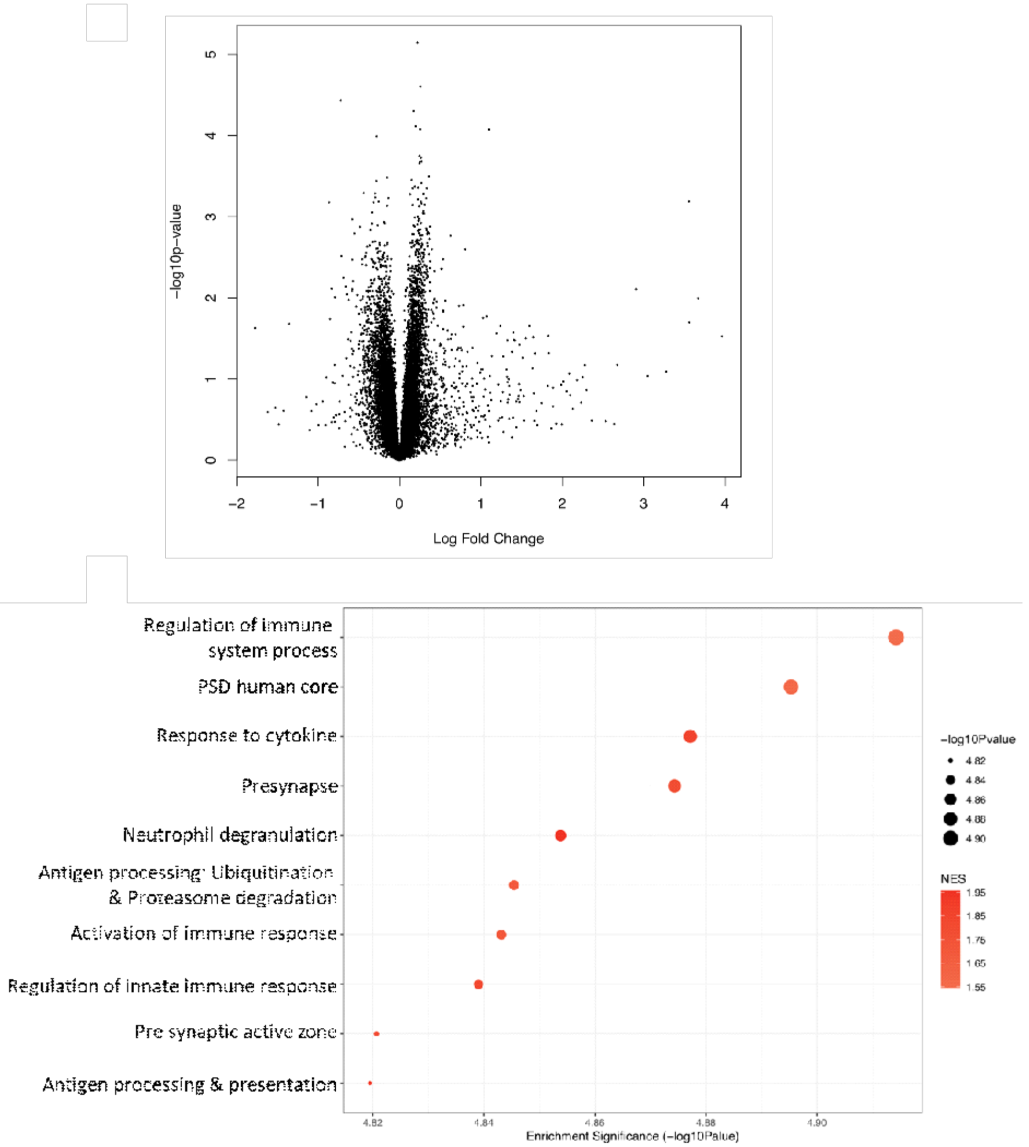

Supplementary Figure 8. Expression differences between NPCs from IL-1β treated versus untreated control NPCs (signature E) at the gene and pathway level. A. The volcano plot shows, on the *y*-axis, the statistical significance (-$\log_{10}$ p-value) of differential expression of genes in IL-1β-treated compared to untreated control cells. The *x*-axis is the magnitude of change ($\log_2$ fold change) in expression of those genes after IL-1β treatment. **B.** The top 10 significantly enriched gene set clusters (the gene set with the lowest *p*-value in each cluster is labelled on the *y*-axis). Data-points are sized here according to significance (-$\log_{10}$ p-value), and coloured according to the normalised enrichment score (NES), with darker red indicating greater upregulation.

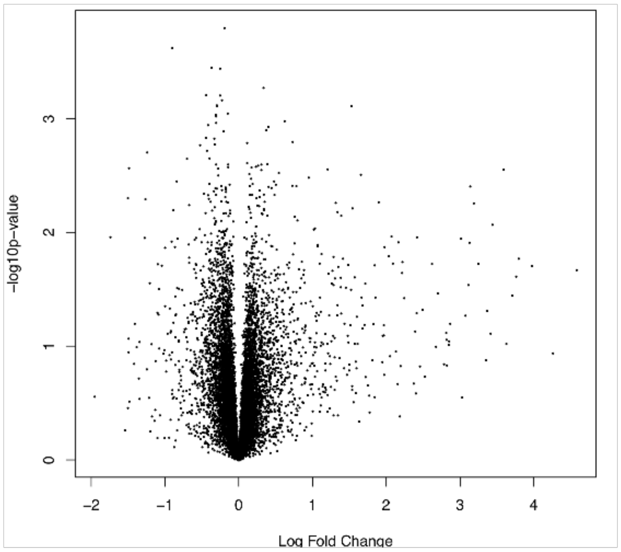

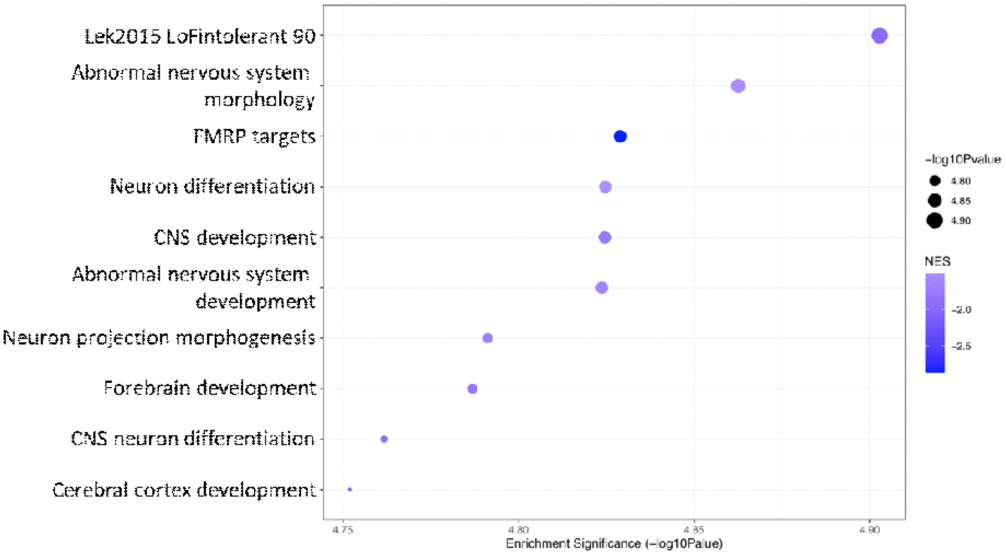

Supplementary Figure 9. Expression differences between NPCs from IL-1β treated versus untreated schizophrenia NPCs (Signature F) at the gene and pathway level. **A.** The volcano plot shows, on the *y*-axis, the statistical significance (-$\log_{10}$ p-value) of differential expression of genes in IL-1β-treated compared to untreated cells from patient (SCZ) donors. The *x*-axis is the magnitude of change ($\log_2$ fold change) in expression of those genes after IL-1β treatment. **B.** The top 10 significantly enriched gene set clusters (the gene set with the lowest *p*-value in each cluster is labelled on the *y*-axis). Data-points are sized according to significance (-$\log_{10}$ p-value) and coloured according to normalised enrichment score (NES), with darker blue indicating greater downregulation.

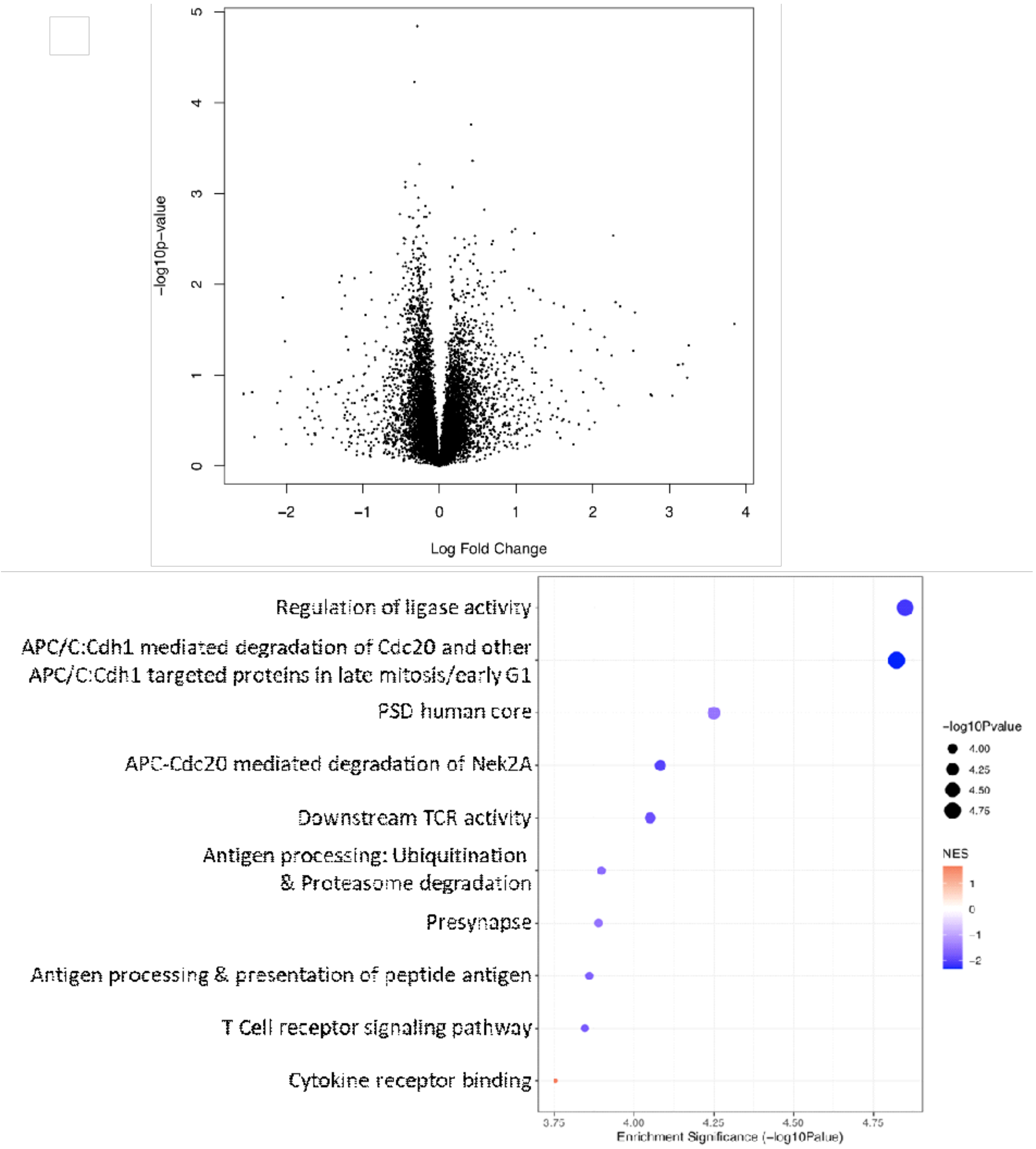

Supplementary Figure 10. Interaction effect between IL-1β-treatment and diagnostic group on gene expression (Signature G). A. The volcano plot shows, on the *y*-axis, the statistical significance (-$\log_{10}$ p-value) of differential expression of genes in IL-1β-treated compared to untreated cells from patient donors versus those from control donors (i.e., the interaction effect between IL-1β -treatment and diagnostic group on gene expression). The *x*-axis is the magnitude of change ($\log_2$ fold change) in expression of those genes after IL-1β treatment. **B.** The top ten significantly enriched gene set clusters

(the gene set with the lowest *p*-value in each cluster is labelled on the *x*-axis). Data-points are sized according to significance (-$\log_{10}$ p-value) and coloured according to normalised enrichment score (NES), with blue indicating downregulation and red indicating upregulation.

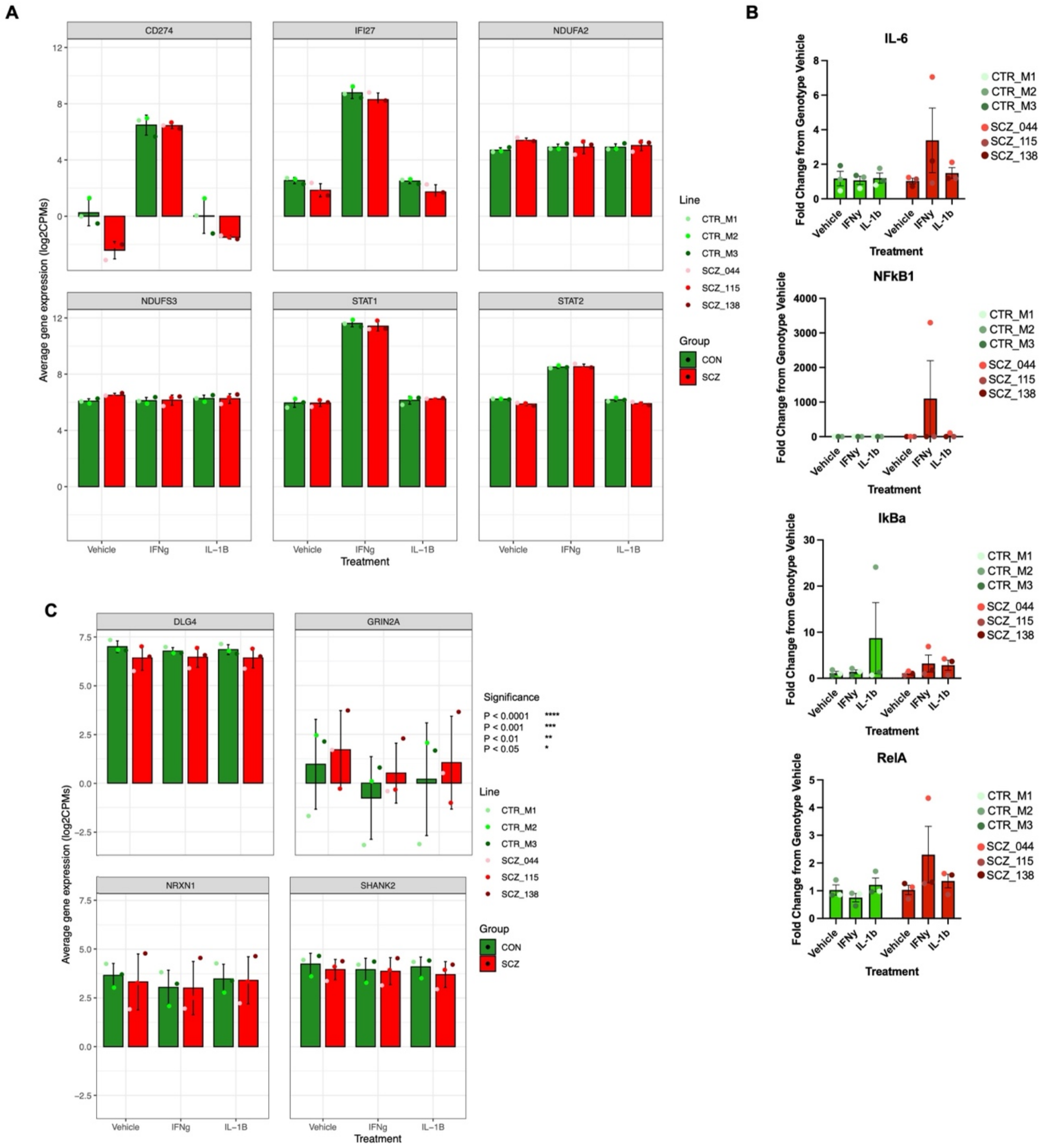

Figure 11*.* Treatment-dependent expression of genes ($\log_2$ CPMs) following IFNγ treatment. A. Gene expression of the most significant DEGs in the differential expression

analysis, for schizophrenia (red) and control (green) lines. **B.** Validation of gene expression changes, or lack thereof, by qPCR. **C.** No change in the expression of synaptic gene assessed were observed.

References

1. Purcell S, Neale B, Todd-Brown K, Thomas L, Ferreira MA, Bender D, Maller J, Sklar P, de Bakker PI, Daly MJ *et al*: **PLINK: a tool set for whole-genome association and population-based linkage analyses**. *Am J Hum Genet* 2007, **81**(3):559-575.
2. Genomes Project C, Abecasis GR, Auton A, Brooks LD, DePristo MA, Durbin RM, Handsaker RE, Kang HM, Marth GT, McVean GA: **An integrated map of genetic variation from 1,092 human genomes**. *Nature* 2012, **491**(7422):56-65.
3. Loh PR, Danecek P, Palamara PF, Fuchsberger C, Y AR, H KF, Schoenherr S, Forer L, McCarthy S, Abecasis GR *et al*: **Reference-based phasing using the Haplotype Reference Consortium panel**. *Nat Genet* 2016, **48**(11):1443-1448.
4. Durbin R: **Efficient haplotype matching and storage using the positional Burrows-Wheeler transform (PBWT)**. *Bioinformatics* 2014, **30**(9):1266-1272.
5. Choi SW, O'Reilly PF: **PRSice-2: Polygenic Risk Score software for biobank-scale data**. *Gigascience* 2019, **8**(7).

6. The Schizophrenia Working Group of the Psychiatric Genomics Consortium R, S., Walters, J.T.R., O'Donovan, M.C.: **Mapping genomic loci prioritises genes and implicates synaptic biology in schizophrenia**. *MedrXiv* 2020

.
